# Caring is not always sharing: A scoping review exploring how COVID-19 containment measures have impacted unpaid care work and mental health among women and men in Europe

**DOI:** 10.1371/journal.pone.0308381

**Published:** 2024-08-30

**Authors:** Hande Gencer, Regina Brunnett, Tobias Staiger, Hürrem Tezcan-Güntekin, Kathleen Pöge

**Affiliations:** 1 Department of Prevention and Evaluation, Leibniz Institute for Prevention Research and Epidemiology–BIPS, Bremen, Germany; 2 Department of Epidemiology and Health Monitoring, Robert Koch Institute, Berlin, Germany; 3 Department of Health Sciences, Fulda University of Applied Sciences, Fulda, Germany; 4 Faculty of Social Welfare, Baden-Wuerttemberg Cooperative State University (DHBW), Villingen-Schwenningen, Germany; 5 Faculty of Health and Education, Alice Salomon University of Applied Sciences, Berlin, Germany; University of Oulu: Oulun Yliopisto, FINLAND

## Abstract

**Introduction:**

Unpaid care work is mainly performed by women, whose mental health is more affected by caregiving burden and work-family conflict compared to men. COVID-19 containment measures may have exacerbated existing gender inequalities in both unpaid care work and adverse mental health outcomes. This scoping review provides an overview of recent evidence on the impact of COVID-19 containment measures on unpaid care work and mental health for subgroups of caregivers at the intersection of gender and other social differences (e.g., ethnicity, age, class) in Europe.

**Methods and analysis:**

Our study was informed and guided by Arksey and O’Malley’s methodological framework. We searched six academic databases (Medline, PsycInfo, Scopus, CINAHL, Social Sciences Abstracts, Sociological Abstracts, ASSIA) and hand-searched the reference lists of selected articles to identify relevant peer-reviewed research articles published between 1 March 2020 and 7 September 2022. In addition, we conducted a grey literature search using Google Scholar and a targeted hand search on known international and European websites. We included studies that reported gender-disaggregated results on unpaid care work and mental health in the context of COVID-19 containment measures in Europe. Two reviewers independently screened all abstracts and full texts for eligibility and extracted the relevant data. The results were synthesised narratively.

**Results and discussion:**

Our results suggest a greater gender gap in unpaid care work division and, to a lesser extent, in mental health, which is unfavourable towards women and mothers. Despite this, we see a break in the traditional division of childcare, with fathers taking on a greater role in family work, which makes us optimistic about the division of care work in the post-COVID-19 era. This research also shows that among European women, population groups often understudied, such as women who are single parents, disabled or of colour, have the highest increase in unpaid care work and greatest deterioration in wellbeing.

## Introduction

The introduction of COVID-19 containment measures, including contact restrictions, closures of workplaces, educational, recreational and cultural, childcare and other care facilities, across Europe from March 2020 resulted in an increased demand for unpaid care work [1], particularly for those with existing caregiving responsibilities towards young children and persons in need of personal care. Unpaid care work can be understood as unpaid services to household members, relatives and friends, including both caring for other people (e.g., childcare, looking after members of the extended family) and reproductive work (e.g., household chores, day-to-day shopping), which are tasks predominantly performed by women [2, 3]. The reasons for the gendered division of unpaid care work are often rooted in cultural and institutionalised gender norms and exacerbated by persistent societal expectations that reproduce an unequal distribution of responsibilities [4, 5]. Additionally, other categories of social differentiation such as age, ethnic origin, migration status, sexual orientation, disability and different living circumstances (e.g., employment status, type of paid work, income, living with a partner and/or children, care arrangements, housing characteristics) can play a role in the uptake and burden of unpaid care work [6].

According to research by the European Union prior to the pandemic, childcare and housework duties were unequally distributed between gender groups [7], with women spending on average more hours per week on unpaid care work than men [8], even among working and non-working coupled parents with young children [9]. A similar pattern is observed for informal caregiving to sick, disabled, or elderly family members or friends, with the greatest gender difference in the 50 to 64 age group (28% of women, 17% of men) [10]. These findings suggest that over their life course, women caregivers are simultaneously or partially exposed to a combined burden of unpaid care work and paid work [11]. Moreover, the gender care gap translates into gender differences in labour market participation: women are more likely to work part-time, which contributes to gender gaps in employment, pay and future pensions [7].

The reconciliation of unpaid care work and paid work is made more difficult by socio-structural factors [12, 13]. Paid work structurally requires relief from the responsibility for unpaid care work and is at the same time a central prerequisite for securing a livelihood, especially in old age [14, 15]. Work-family conflicts along with long and delimited working hours can have a negative impact on the mental health of unpaid caregivers [16–18]. Across European countries, informal caregivers report lower levels of mental wellbeing compared to non-caregivers, especially women and intensive caregivers [19–21].

Policy measures to contain the spread of the COVID-19 virus could have exacerbated existing gender inequalities in unpaid care work and mental health. The results of Eurofound’s COVID-19 online survey show that compared to men, working and non-working women spent almost twice as many hours per week on unpaid care work, including childcare (12.6 vs. 7.8 hours), informal caregiving (4.5 hours vs. 2.8 hours), housework and cooking (18.6 hours vs. 12.1 hours) [22–24].

Early research findings from Europe suggest a differential impact of COVID-19 containment measures by ethnicity and socio-economic status. In Germany, COVID-19 outbreaks were more common in neighbourhoods with a higher proportion of migrants [25]. In the UK, racialised and migrant population groups were more likely to experience economic difficulties [26], a greater deterioration in subjective wellbeing [26, 27] and a higher death rate after testing positive for COVID-19 compared to white people [28]. Migrant population groups are more likely to work in lower-paid jobs and as precarious essential workers [29]. They may have been less affected by COVID-19-related furlough measures, layoffs, and loss of earnings [30], but are at a higher risk of contracting the virus. In Germany, people working in elderly care, healthcare and nursing–mostly women with a migration history in the EU [7]–were particularly affected by COVID-19-related sick leave compared to other occupational groups [31]. Living in high-density households and chronic illnesses are risk factors for lower subjective wellbeing during the COVID-19 pandemic [32]. These factors are more likely to apply to migrant and lower-income population groups [29].

The aim of this research study was to map the evidence on the gendered and intersectional impact of COVID-19 containment measures in Europe in relation to unpaid care work and mental health. According to the concept of intersectionality, one’s social location is influenced by interlocking systems of privilege and oppression (e.g., (hetero-)sexism, classism, ableism, racism, ageism) that are not simply additive, but interact in complex and uneven ways [33]. Gender inequalities need to be addressed at the intersection of other categories of social differentiation (e.g., ethnicity, immigration status, age, and economic position), as intersections of social locations may increase the risks for adverse mental health outcomes for subgroups of unpaid caregivers. An intersectionality-informed approach enables researchers and policymakers to understand the social and economic consequences of COVID-19 containment measures for women, men, and gender-diverse individuals, including where vulnerabilities intersect and where they diverge [34]. We expect that COVID-19-related containment measures will have had differentially impacted unpaid caregivers at the intersection of gender and other social locations. These intersecting social locations (e.g., being a middle-class migrant mother) cannot be understood as homogenous groups. Therefore, other factors such as socio-economic characteristics (e.g., employment status, working hours, housing situation), living circumstances (e.g., living with a partner, living with young children), and public and labour market policies (e.g., provision of public childcare, long-term care arrangement, reconciliation measures) need to be considered. These influencing factors may have affected the way in which policy measures impact caregivers’ mental health. In addition to these social and systemic factors, caregiving characteristics (e.g., the type of unpaid care work, intensity of caregiving, relationship with the care recipient, absence/presence of illness or disability of the care recipient, living with the care recipient) may have mitigated the impact of unpaid care work on mental health in the context of COVID-19 containment measures.

The COVID-19 pandemic is a very recent phenomenon that was officially declared over by the WHO in early May 2023 [35]. It provides important lessons for future public health crises and policy responses. We decided to conduct a scoping review to explore the scientific literature on the potential adverse impacts of COVID-19 containment measures on the mental health of subgroups of unpaid caregivers. Following World Health Organization’s definition, we defined mental health as the state of wellbeing in which a person realises their own abilities, can cope with the normal stresses of life, can work productively and is able to make a contribution to their community [36]. A preliminary search for similar studies was conducted by hand-searching scientific registers, selected databases and Google Scholar for unpublished and published systematic and scoping reviews on the topic. To our knowledge, this scoping review is the first to identify and map evidence of gender differences in unpaid caregiving and related adverse mental health outcomes in the context of COVID-19 containment measures in Europe.

The aim of the study was to map the current state of research on gender differences in the impact of changes on unpaid care work and caregivers’ mental health in the context of COVID-19 containment measures, in particular to:

1. Identify changes in the distribution of unpaid care work between gender groups under COVID-19 containment measures.
2. Describe the impact of these changes on the mental health of different subgroups of caregivers.
3. Identify population groups particularly affected by pandemic restrictions and changing demands on unpaid care work.
4. Provide recommendations for future public health research and potentially useful gender-equality measures for the post COVID-19 pandemic period and in anticipation of future public health crises.

## Methods

This scoping review consists of a systematic academic database search, complemented by a grey literature search in Google Scholar and a targeted hand search of relevant websites. The database search was conducted according to the methodological framework for scoping studies proposed by Arksey and O’Malley [37] and its extension by Levac et al. [38]. The Preferred Reporting Items for Systematic Reviews and Meta-Analyses extension for Scoping Reviews (PRISMA-ScR) were used to ensure the rigour and replicability of the scoping review (S1 Table) [39]. The identification of the research questions underlying our scoping study and the reasons for limiting our study to the geographical region of Europe are detailed in a published study protocol [40].

### Literature search

The databases Medline, PsycInfo, Scopus, CINAHL, Sociological Abstracts, Social Services Abstracts and Applied Social Science Index & Abstracts (ASSIA) were searched for peer-reviewed research articles published between 1 March 2020 and 7 September 2022. We searched for research articles reporting gender-disaggregated results on mental health outcomes related to unpaid care work in the context of COVID-19 containment measures in Europe. An initial database search was conducted on 21 October 2021 and updated on 7 September 2022 using the same search strategy according to Bramer and Bain’s [41] guide for updating search strategies for systematic reviews. To this end, we re-ran the database searches and imported all results into EndNote 20 software (Thomson Reuters, USA). These were copied into a new EndNote file, and the original results were checked for completeness and duplicates removed. The reference lists of selected articles were hand-searched to identify additional relevant studies. Language limitations were not applied. The search results were exported and uploaded to EndNote and then imported into Covidence [42] for the original search and to Rayyan [43] for the updated search and screening. In addition, a search was conducted in Google Scholar in May 2022. It was not updated as most of the references retrieved were identified via other sources. This search identified relevant grey literature and was conducted in addition to a targeted hand-search on websites of international and European organisations and institutions, which was performed in April 2022 and updated in December 2022. The results of these searches were transferred into Excel spreadsheets for the screening. A detailed documentation of the grey literature search including a guide for a systematic literature search on Google Scholar created by the corresponding author is available in S1 Appendix.

### Search strategy

The database search strategy consisted of a combination of key words and controlled vocabulary (i.e., MeSH-terms, subject headings, and thesaurus words) derived from the search themes (i) “unpaid care work”, (ii) “COVID-19 containment measures”, and (iii) “mental health outcomes”. Titles and abstracts were searched for themes (i) and (ii) which were combined with the Boolean operator AND; search terms of theme (iii) were initially defined to narrow down a potentially high number of hits, but were ultimately not used throughout the process. Further details on the search strategy and search terms can be found in the published study protocol [40]. An example of the search strategy used in the MEDLINE database (via Ovid) can be found in S2 Table. The search terms for Google Scholar were adapted to the database search strategy as described in S1 Appendix.

### Study selection

After removal of duplicates, the remaining identified peer-reviewed research articles and grey literature were screened for eligibility using pre-defined inclusion and exclusion criteria (Table 1). In short, original research articles and grey literature were eligible if they analysed primary or secondary data from European countries collected after the onset of the COVID-19 pandemic in March 2020.They were required to report mental health outcomes and measure unpaid care work, which would enable a description of the differential impact of COVID-19 containment measures by gender. Records from the database and grey literature searches were screened by two independent researchers in two steps: first, titles and abstracts were screened (for the grey literature, the introduction or first two pages were screened where an abstract was missing), and second, the full-texts of included titles/articles were screened. Disagreements between two reviewers were discussed bilaterally and, if necessary, between all reviewers until a consensus was reached.

**Table 1:** Inclusion and exclusion criteria.

*Inclusion criteria*	
1	Publication status: Research articles must be published in a peer-reviewed journal as original research from 1 March 2020 to search date. Grey literature must be published as a report, brief, discussion/ working paper, or book chapter from 1 March 2020 to search date.
2	Study design: Empirical studies with any design except for reviews or clinical studies.
3	Population: Adults (18+) who provide unpaid and non-professional care work.
4	Exposition: Any type of COVID-19 containment measures including (retrospective) pre- and during pandemic measures.
5	Comparison: Unpaid care work and mental health outcomes must be reported by gender to allow for between-gender comparison.
6	Outcomes: Any type of mental health measures including indicators of mental wellbeing (e.g., subjective wellbeing, aspects of life satisfaction, happiness), mental disorders (e.g., diagnoses of depression, schizophrenia, burnout, anxiety disorders; self-reported (symptoms of) mental disorders, use of mental health services, use of medications for mental disorders; help-seeking behaviour regarding mental health problems, number of medical referrals for treatments of mental disorders; self-reported limitations in daily activities due to mental disorders; substance abuse including alcohol abuse) and perceived caregiver burden.
7	Setting: Europe.
*Exclusion criteria*	
1	Wrong article type (e.g., conference abstracts, pre-prints, letters, editorials, comments, book reviews, monographs, recommendations, calls for action, opinion pieces, blog entries, news, theses).
2	Wrong study type (systematic literature reviews, clinical studies, literature reviews without original data).
3	Wrong population (other target population than adult unpaid caregivers, e.g., care receivers, minors) or population not defined.
4	Wrong exposition (no mentioning of COVID-19 containment measures).
5	Wrong comparison (no gender-disaggregated data for unpaid care work and mental health).
6	Wrong outcome (no assessment/ report of mental health outcomes).
7	Wrong setting (no reports of European data).

### Data extraction

Data extraction was limited to the relevant data items shown in Table 2; additional information and results that were irrelevant to the research questions were disregarded. Data from the full texts were extracted by two researchers independently who compared their findings before discussing them within the research team. Further information on data extraction and charting can be found elsewhere [40]. As it is not a common practice to assess the quality of studies in scoping reviews, the quality of selected studies was not formally appraised. However, reported study limitations were extracted and summarised together with the limitations identified by the research team in S3 Table.

**Table 2:** Data items extracted from included full texts.

1	Bibliographic information (first three authors, publication year, title, setting, publication type)
2	Study details (research methods, study design, data source, data collection, sampling, study period, objectives/focus)
3	Population details (sample size and relevant demographics by gender)
4	Measurement of COVID-19 containment measures
5	Measurement of unpaid care work
6	Measurement of mental health
7	Relevant health-related measures
8	Key findings on unpaid care work and mental health outcomes related to COVID-19 containment measures by gender and by subgroups
9	Observed predictors/ mediators
10	Study limitations
11	Conceptualisation of sex/gender
12	Degree of intersectionality (direct, indirect, or no mentioning; article or report part where intersectionality was mentioned; relevant theory/ power dynamics; social categories)
13	Conceptualisation of gender & gender inequality

### Data analysis

The extracted data was coded into quantitative categories, qualitative descriptions or authors’ statements and documented in Excel spreadsheets, which served as data charting forms. This formed the basis for the narrative and descriptive syntheses of relevant measures and characteristics. The results were described narratively and reported along the main categories of the research questions.

### Patient and public involvement

Neither patients nor members of the public were involved in the development of this scoping review. However, we do recognise that participatory research methods, such as consulting the target population in the development of research questions and the discussions of results, would have led to a much more realistic representation of the lived realities of people with unpaid caregiving responsibilities.

## Results

### Search and study selection

**Fig 1:**
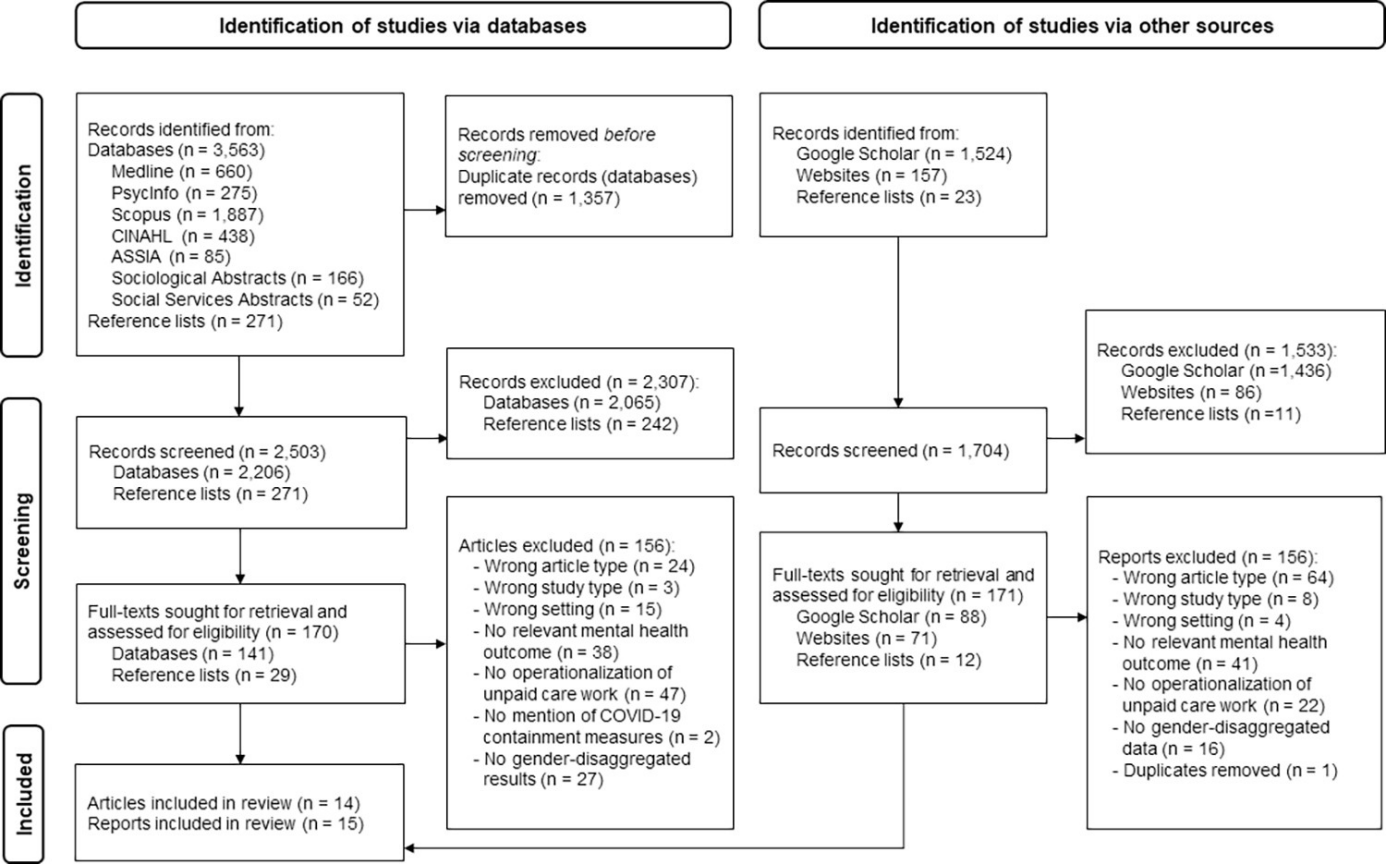
PRISMA flow chart of study selection process.

The database search yielded 3,563 potentially relevant records and 271 additional records that were identified via hand-searching reference lists. After removing duplicates, 2,503 titles and abstracts were initially screened; 2,307 articles did not meet the inclusion criteria, leaving 170 full-text articles for review. A total of 14 research articles were selected for inclusion. The grey literature search yielded 1,704 references, of which 1,524 were identified via Google Scholar, 157 via websites and 23 via reference lists. Ultimately, 171 full texts were screened, of which 15 reports were included in this review. This scoping review reports on 29 studies that met the inclusion criteria. The study selection process is illustrated in Fig 1.

### Study characteristics

The key characteristics of the included studies are summarised in Table 3 and S3 Table. Settings included the UK (n = 10), Germany (n = 9), Italy (n = 2), Austria (n = 1), the Netherlands (n = 1), Slovenia (n = 1), Spain (n = 1), Türkiye (n = 1) and Europe (n = 3). Most of the articles and reports used quantitative research methods (n = 26), with only two qualitative studies and one mixed-methods study. Data was collected through surveys and interviews conducted exclusively online and/or by telephone. Seventeen studies used convenience samples and 12 studies used nationally representative datasets.

**Table 3:** Study characteristics of included literature.

Author(s), year, setting, study period	Objectives	Unpaid care work definition	Mental health definition	Sample & Demographics	Methods
*Peer-reviewed research articles*					
Ashencaen Crabtree et al., 2021 [44] UK Mid-April to 12 June 2020	To identify factors to improve academics’ work-life balance during and after lockdown in the UK and beyond.	Domestic responsibilities including childcare, home-schooling, and, to a lesser degree, care responsibilities toward sick, disabled, or old adults.	General quality of life (work-life balance and related stress, changes in workload and leisure time, concerns regarding the end of lockdown).	N = 146 academics living in the UK, with n = 114 women (predominantly senior lecturers and lecturers from the disciplines of social sciences and humanities & medical sciences and nursing) and n = 29 men (predominantly professors and senior lecturers from the disciplines of natural sciences and engineering & social sciences and humanities).	Descriptive statistics & chi-square test of independence to assess dependency of responses on gender for quantitative data; narrative analysis of qualitative data.
Balenzano et al., 2020 [45] Italy 18 May—15 June 2020	To explore the effects of social isolation on Italian families with small children experienced during lockdown.	(1) Perceptions of equal division of domestic work and parenting tasks (“work-family balance”) between gender groups before and during lockdown (4-point Likert rating scale (from 0 = not at all to 3 = very much); (2) indication of who took over primary responsibility of young children during phases of lockdown (categorical answers).	(1) Satisfaction with division of a) domestic work and b) childcare responsibilities within families (4-point Likert rating scale (from 0 = not at all to 3 = very much); (2) parenting stress: parents were asked how much, in their perception, emergency restrictions and the resulting social isolation had increased their parenting stress (4-point Likert rating scale (from 0 = not at all to 3 = very much).	N = 104 parents of intact families with at least one preschool or school-aged child (under 14 years) living in Italy, with n = 84 mothers (mean age 40.41) and n = 20 fathers (mean age 42.5).	Descriptive statistics & combinations of chi-squared test, ANOVA, frequency analysis and paired t-test to explore pre-post-lockdown and possible gender differences in outcome variables.
Bartolj et al., 2022 [46] Slovenia 16 March– 18 May 2022	To analyse differences in lockdown effects on time allocation and emotional exhaustion between parents and non-parents.	Self-reported proportion of awake time allocated to unpaid care work (as well as leisure and paid work) and hours of sleeping amid/before the COVID-19 pandemic, with hours devoted to each activity being calculated as “(proportion of awake time allocated to activity) x (24 –h of sleep)”.	Emotional exhaustion measured with three questions comparing the prevalence of emotional drain, fatigue (when getting up in the morning and having to face obligations of another day), and work strain during the COVID-19 pandemic compared to the pre-pandemic period; for each question, 0 was assigned where there was equal frequency, negative values for lower frequencies, and positive values for higher frequency during COVID-19 compared to before; based on these three values, a variable “emotional exhaustion” was calculated where higher scores indicate a greater increase in emotional exhaustion.	N = 1,231 employees in partnerships where both partners were teleworking or working remotely from home, with 71% women, 24% being 35 years or younger, 62% living in an urban area, and 59% parents.	Frequency analysis; Difference-in-Difference (DID) method.
Beno, 2021 [47] Austria Not reported	To investigate the situation of face-to-display working parents with school-age children facing various face-to-display work and school challenges, especially when schools are closed.	Responsibilities of additional childcare tasks due to lockdown; balancing working from home and home-schooling.	General wellbeing with regards to working from home during closures of schools and care facilities.	N = 10 full-time face-to-display working parents (services sector) of school-aged children, with n = 5 women and n = 5 men and an age range of 37–51.	Analysis of interview transcripts by means of two-level coding scheme.
Cannito & Scavarda, 2000 [48] Italy March—May 2020	To investigate the consequences of remote work on work-life-balance and gender inequalities in the division of paid and unpaid labour within heterosexual couples.	Childcare involvement (educational, caring); work-life balance.	Expressions of (increase/ decrease in) stress, anxiety, worries, sense of guilt, self-confidence related to work-life balance during lockdown; satisfaction with paid work, care work and work-life balance.	N = 10 heterosexual couples working from home with children under the age of 11 living in Northern Italy, with n = 10 women and n = 10 men (high education, good socio-economic position).	Thematic analysis of interview transcripts.
Cheng et al., 2021 [49] UK Three waves: 2017–18, April & May 2020	To explore the connection between financial security, working from home, and childcare with regards to the COVID-19 pandemic and related containment measures among working parents.	Time-use on childcare and home-schooling with four intensity categories according to weekly hours spent on these activities with (1) less than 1 hour, (2) 1–7 hours, (3) 7–20 hours, (4) 20 hours + per week.	GHQ-12^1^ Caseness score with ranges 0 (the least distressed) to 12 (the most distressed).	N = 6,795 working parents (employed or self-employed) living with at least one child younger than 18 (58% women, 86% White, mean age 43.1) and N = 8,870 individuals who work (employed or self-employed) as comparison group.	Descriptive statistics & OLS estimations with separate regressions by type of working parents as explanatory variable of interest.
Clemens et al., 2021 [50] Germany 18 May—21 July 2021	To assess the role of ACEs and other sociodemographic factors associated with potential harmful parenting actions and parental coping of the current pandemic.	Childcare during the pandemic (“Who took care of the child during most of the time during the Covid-19 pandemic?”) with answer categories mainly by oneself, mainly by partner, mainly equally by oneself and partner, mainly by someone else.	Satisfaction with childcare arrangements (“Please indicate on a scale of 0–100 how satisfied you are with the sharing of childcare duties between you and your partner (before the pandemic and now)?”), with 0 being the worst and 100 being the best conceivable satisfaction.	N = 687 parents of a minor, with n = 15 women (mean age 41.1, 58% high education, 52% working from home) and n = 72 men (mean age 45.8, 78% high education, 53% working from home).	Linear regression analyses to identify factors associated with parenting and successful coping of pandemic-related challenges; two-way repeated measures ANOVA was used to test differences in parental satisfaction with the sharing of childcare duties before and during the pandemic.
Czymara et al., 2021 [51] Germany 27 March—26 April 2020	To study the impact of COVID-19 by examining main experiences, concerns, and worries during first weeks of lockdown and assessing whether these experiences varied by gender.	Cognitive labour (i.e., anticipating needs and identifying options for meeting those needs); looking beyond household work to a more holistic view of work (regardless of whether paid or unpaid, done inside or outside home); general family life.	Mental load (i.e., concerns, worries, personal experience); “anticipation needs” and “identifying options” as part of cognitive labour.	N = 1,119 oversampled young and highly educated individuals, 63% at least university degree, 58% younger than 45, with n = 884 women and n = 235 men.	Mixed-methods approach consisting of (1) structural topic modelling (inductive machine-learning approach) to detect topics of concern and statistically test gender difference in concerns and (2) qualitative analysis of open survey questionnaire.
Giurge et al., 2020 [52] Spain^2^ Mid-March to mid-June 2020	To explore how different groups of people spent their time during the pandemic, and whether and how time use shaped their subjective wellbeing.	Time spent on “necessities” including (1) household chores (e.g., preparing meals, doing laundry, cleaning) and (2) taking care of others/ family members measured as number of weekly hours; necessities composite used in analyses.	SWB by asking participants to rate their overall happiness on a scale from 0 (not at all) to 10 (extremely).	N = 975 public sector workers in Spain (mean age 47.0), with 69% women, 89% parents, 44% masters’ degree and above.	Originally mega- and meta-analyses pooling multiple survey samples. Sub-sample regression analyses.
Hipp & Bünning, 2021 [53] Germany Three waves between 23 March and 2 August 2020^3^	To examine the social and economic implications of the COVID-19 pandemic for gender inequality in Germany by exploring changes in paid and unpaid work as well as in subjective wellbeing in response to containment measures.	Division of housework (1) and division childcare (2) among couples with three response categories “I do all/most of the work”, “equal sharing between partners”, and “my partner does all/most of the work”.	Satisfaction with work, family life, and life in general (adapted from the SOEP, measured on a 7-point Likert scale, not further defined).	N = 4,429 persons who participated in all three waves aged 25–54, with n = 3,391 women.	Regression analyses
Ohlbrecht & Jellen, 2021 [54] Germany 14 April—3 May 2020	To assess psychosocial consequences of COVID-19 containment measures.	Response to question “To what extent do the following possible situations or life circumstances apply to you in the course of the corona pandemic? a) I am increasingly responsible for the care of underage persons due to the closure of facilities (day care centre, school, etc.). b) I have to provide schooling for my children predominantly on my own.” on a 5-point rating scale (1) not applicable at all 2) rather not applicable 3) moderate 4) rather applicable 5) fully applicable 6) not applicable to me).	Subjective wellbeing before and after the COVID-19 outbreak based on three indicators: (1) frequency indications of feelings of nervousness, exhaustion, security, satisfaction, happiness, loneliness, stress, fear, fear of existence, sadness, serenity with answer categories 1) “never” 2) “rarely” 3) “sometimes” 4) “often” 5) “very often” (categories 4 and 5 were merged); (2) self-rated life satisfaction with answer categories 1) “very unsatisfied” 2) “unsatisfied” 3) “moderate” 4) “satisfied” 5) “very satisfied” 6) “not specified” (categories 1 and 2, 4 and 5 were merged); (3) perception of stress during the pandemic (“When you look back on the lockdown so far, how much strain did you feel?”) with answer categories 1) “not at all” 2) “a little” 3) “moderate” 4) “strong” 5) “very strong” (categories 1 and 2, 4 and 5 were merged).	N = 2,009 persons with 71% women, ca. 1/3 aged under 30 and 1/3 aged 30–40, 95% born in Germany and 82% with higher education entrance qualification, and 80% employed.	Frequency analysis, descriptive analyses of participant information for quantitative data; open questions analysed with coding system.
Xue & McMunn, 2021 [1] UK Three waves: 2017–19, April 2020, May 2020	To describe gender divisions of unpaid work during the height of the COVID-19 lockdown in the UK and its associations with psychological distress.	Time-use on housework and childcare or home-schooling in number of hours in the last week. Additionally, division of childcare within couples and reduction or adaptation of employment hours to accommodate childcare, responses being “neither”, “both”, “mother only”, or “father only”.	GHQ-12^2^ Likert score	Six samples with four interviewed at two time-points: All participants (April: 13,218 with 68% women; May: 12,472 with 66% women), couples (April: 7,009; May: 5,656), parents (April: 4,174; May: 3,179), couple parents (April: 1,731; May: 1,551), working parents (April: N/A; May: 2,990), and working couple parents (April: N/A; May: 1,572)	Descriptive statistics, linear regression models (complete case analysis) to assess association between unpaid care work and GHQ Likert scores, and sensitivity analyses to investigate the extent to which gender differences in unpaid care work remained after adjusting for demographic differences.
Yerkes et al., 2020 [55] Netherlands 13 April—28 April 2020	To explore the differences between mothers and fathers in paid work, the division of childcare and household tasks, and quality of life, and whether changes take place in these dimensions comparing pre-lockdown to during lockdown situations.	Respondents indicated, relative to their partner, how much (1) housework and (2) caregiving tasks (incl. home-schooling and help with homework) they did prior and during lockdown with a 7-point response scale ranging from 1 = “I do nearly everything” to 7 = “My partner does nearly everything”; based on a pre- and during-lockdown comparison, two dummy variables indicating a) whether the relative share of the respondent had increased and b) whether the relative share had decreased were computed separately for (1) and (2).	Three dimensions of self-assessed quality of life: (1) leisure (originally 5-point response scale recoded into three categories (much) less leisure time (a combination of much less and slightly less leisure time), no change in leisure time (= reference category), and (much) more leisure time (a combination of slightly less and much more leisure time)), (2) work-life balance (adapted from EUROFOUND Quality of Life survey, two questions asking respondents to report on how easy or difficult it was to combine paid and unpaid care work prior to and during lockdown, with 5-point Likert scale ranging from 1 = very easy to 5 = very difficult), (3) relationship dynamics (respondents indicated how often they had disagreements with their partner prior to and during lockdown about five issues: work location, scheduling of working hours, housework, caring for children, and leisure time. Answer categories included (1) never, (2) monthly, (3) sometimes, (4) weekly and (5) almost daily; change from before to during lockdown was measured by indications of the frequency of disagreements on the same five issues compared before and during lockdown, measured on a 5-point scale ranging from (1) a lot less often to (5) a lot more often recoded into two dummy variables ’less conflict with my partner’ (yes/no) and ’more conflict with my partner’ (yes/no)).	N = 852 respondents in a household with at least one member in paid employment and at least one child under the age of 18 living at home, with 56% women and a mean age of 43.	Descriptive methods (cross-tabulation) and multivariate modelling.
Zhou & Kan, 2021 [56] UK Ten waves between 2017 and 2021^4^	To examine how the spread of COVID-19 and COVID-induced policies have had unequal and dynamic impacts on different social groups in the UK.	Time-use on a) housework and b) childcare in weekly hours (“Thinking about last week, how much time did you spend on housework, such as time spent cooking, cleaning and doing the laundry?” and “About how many hours did you spend on childcare or home-schooling last week?”, respectively.)	GHQ-12^2^ Likert score	N = 78,307 individuals of prime age (20–65), weighted data.	Linear fixed-effect regression models interacting the month indicator with gender, BAME group, and education levels, to examine how the change in income, time use, and wellbeing differed across individuals in the three different sociodemographic groups in different periods of the pandemic.
*Grey literature*					
Bolis et al., 2020 [57] GB^10^ May–June 2020	To explore how COVID-19 and related lockdown measures have affected women’s and men’s unpaid care workloads, how this varies across different contexts by race, ethnicity, income, age, and the type of household (single or dual parents), and the impact this is having on health, economic security, and wellbeing.	Categorical answers to the question whether an increase in care and domestic work was recorded through estimated ranges (e.g., up to two hours, two-three hours, etc.); no further specification.	An affirmative answer to the question whether one was feeling more anxious or depressed because of increased unpaid care and domestic work due to the pandemic.	N = 1,662 respondents (n = 808 men, n = 854 women) aged 18 and over; weighted data used for analyses.	Descriptive statistics; frequency analysis.
Bujard et al., 2020 [58] Germany 17–23 April 2020^5^	To explore the impact of the Corona crisis on parents’ family and work lives.	Time-use on family work in hours per regular weekday based on the question “How many hours did you spend on childcare, education of children, informal sick/elderly care and housework on an average weekday in the last seven days?” with focus on childcare.	Aspects of life satisfaction based on the question “How satisfied are you currently with the following life domains: Work life… family life…” on a 11-point scale ranging from 0 = not at all satisfied to 10 = completely satisfied. Average points of 7 and above indicate high life satisfaction, average points below 6 (less common) indicate great dissatisfaction.	N = 2,024 employed persons with n = 1,708 men and n = 946 women aged 16–75; Weighted data used for analyses.	Descriptive statistics; Frequency analysis.
Chung et al., 2020 [59] UK 22 May- 15 June 2020	To gain insights on how work and home-life have changed throughout the lockdown period and how these changes might have a profound impact on both the future of work and gendered cultural norms around care in the UK.	Perceptions of the division of housework and care: Those living with a significant other were asked how they divided up housework activities distinguished into six categories including: cooking, cleaning/laundry, DIY (home, garden, care maintenance etc.), routine childcare (generally looking after children), non-routine childcare (playing with and entertaining children), and children’s education (supervising homework and home learning during the lockdown). Answer categories included: a) women carry out most/at of the housework/care and b) both parents share, or men partners do most of the housework/care.	Wellbeing: reports of feeling (1) rushed or pressed, (2) nervous and stressed (no further specification), (3) cheerful and in good spirits, (4) calm and relaxed, (5) active and vigorous, (6) fresh and rested, (7) life interesting more than half of the time (including most/all time) in the past month (during lockdown).	N = 1,161 heterosexual coupled employees with children aged under 18 with n = 648 mothers, n = 236 fathers, n = 199 women and n = 78 men who did not live with a child under the age of 18.	Descriptive statistics
Close the Gap & Engender, 2021 [60] Scotland 8 Nov.– 2 Dec. 2020	To look at the implications of the COVID-19 crisis on women with childcare responsibilities in Scotland.	Frequency indications regarding (1) housework (e.g., cleaning and laundry) and (2) shopping for groceries and essentials (mostly done by oneself, mostly done by one’s partner, both equally).	Indication of experienced anxiety during the COVID-19 crises as a response to the question “Overall, on a scale of 0–10, where 0 is not at all and 10 is completely, how anxious did you feel yesterday?” with a response between 6 and 10 indicating a high level of anxiety.	N = 721 parents with children aged 14 and under.	Frequency analysis
Destatis, WZB & BiB, 2021 [61]Click or tap here to enter text. Germany April 2020	No objectives provided.	Time spent on house and family work in daily hours.	Satisfaction with work arrangement and family life using a 10-point scale ranging from 1 = “not at all satisfied” to 10 = “very much satisfied”.	Not reported; weighted data used for analyses.	Frequency analysis
Etheridge & Spantig, 2020 [62] UK Two waves: 2017/18, 24–30 April 2020	To examine which social factors explain the gender gap in wellbeing on aggregate.	Self-reported time use on childcare (0 h, 1–15 h, 15+ h), and housework (<6 h, 6–10 h, 10+ h) in the previous week (weekly hours in three categories); dummy variable for caring duties (responsibilities of caring for somebody outside the current residence in the previous 4 weeks, yes/no).	GHQ-12^2^ Likert score	N = 12,250 respondents; weighted data used for analyses.	Frequency analysis; correlation analysis; regression analysis.
Eurocarers/ IRCCS-INRCA, 2021 [63] Europe^6^ 24 Nov. 2020–8 March 2021	To analyse the impact of COVID-19 on informal carers’ health, caregiving situation, support networks, access to health and social services, working status, work-life balance and finances and collect their views and recommendations how to better support them during the pandemic.	Average number of weekly hours of informal care provided.	Frequency indications of aspects of carers’ lives that were impacted by COVID-19 including “mental health/psychological state of mind”; no further specification of operationalisation.	N = 2,468 European informal carers who provided regular care (i.e. not occasional or temporary) to one or more people with their daily activities, personal care or in any other way due to their physical or mental illness, disability or old age; with 80% women and a mean age of 57.3 years, 88% highly educated respondents (at least 9 years of schooling) and 50% university degree, 74% married or cohabitant, 63% co-residing with care recipient.	Descriptive statistics; frequency analysis.
Eurofound, 2020 [22] EU-27 April & July 2020	To capture the immediate impact of the pandemic on the way people in Europe live and work.	Time-use in hours over the past month on a) caring for children and grandchildren, and b) doing household work	WHO-5 mental wellbeing index measuring people’s moods over the previous two weeks based on five statements of positive feelings (“I have felt cheerful and in good spirits”, “I have felt calm and relaxed”, “I have felt active and vigorous”, “I woke up feeling fresh and rested”, “My daily life has been filled with things that interest me”); measured on a scale from 0 to 100; feeling lonely in the previous two weeks with answers being all or most of the time among others (not further specified).	N = 91,753 global participants (N = 87,744 EU27).	Descriptive statistics; frequency analysis.
Hübgen et al., 2021 [64] Germany/Berlin Four waves in 2020/ 2021^7^	To examine the impact of the COVID-19 pandemic on the economic and social lives of women in Berlin.	Answer to the questions of who is responsible for (1) childcare and (2) housework with response categories being predominantly oneself, half/half, predominantly one’s partner.	Frequency indications of experiencing feelings of anxiety/ nervousness/ restlessness and hopelessness/ stress on a 5-point Likert scale ranging from 1 = never to 5 = all the time; aspects of life satisfaction (work, family life, general life) on a 7-point scale ranging from 1 = very unsatisfied to 7 = very satisfied.	(t1): N = 9,492, (t2): N = 7,573, (t3): N = 6,397, (t4): N = 6,472; main analyses with respondents aged 18–59 with 72% women, 47% of persons living with at least one minor and 72% academics at wave 1.	Logistic regression analysis
Illing et al., 2022 [65] Germany Five waves in 2020/ 2021^8^	To study the change in time allocation among employed women and men (liable for social insurance) at the beginning for the COVID-19 pandemic.	Time in weekly hours spent on childcare.	Life satisfaction measured on a 5-point Likert scale (no further specification).	N = ca. 11,000 persons employed in December 2019 in jobs with social insurance liability and employed in previous years, aged 24–55, ca. 50/50 women and men; weighted data used for analyses.	Event study with person-fixed-effects regression
Kalaylıoğlu et al., 2020 [66] Türkiye 18–25 April 2020	To explore the differential short-term impact of the COVID-19 crisis and related measures on the socio-economic status of men and women both at work and at home in Türkiye.	Change of the number of hours devoted to various household activities as a result of COVID-19 with four answer categories (“I do not usually do it”, “increased”, “unchanged”, “decreased”); indication of one household activity among 11 categories participants spend the most time in since the spread of COVID-19; indication in how far household roles and responsibilities within families (eight areas in total) have been affected since the spread of COVID-19 with three answer categories (“yes”, “no”, “not applicable”).	Personal experience of psychological/ mental/ emotional health (e.g., stress, anxiety, etc.) being affected as a result of COVID-19 with three answer categories (“yes”, “no”, “not applicable”).	N = 1,508 men and women aged over 15, with n = 759 women (51.1% married) and n = 749 men (63.6% married) with an average age of 38.7, 30% living with children under 18 in their household (average household population of 3.46 people), 21% university degree (19.1% of women vs. 23.4% of men), 37% primary-secondary school graduates and 39% high-school graduates (3.8% of women vs. 1.1% of men).	Frequency analysis
OECD, 2021 [67] OECD countries^9^ Last quarter of 2019 & third quarter of 2020	To illustrate cross-national comparisons of the effects of the COVID-19 induced recession on (working) mothers.	Perception of who took on more unpaid childcare work as response to the question “In your household, who took on any additional care work as a result of school or childcare facility closures [during COVID-19]?” with response options being “entirely you”, “mostly you”, “equally shared between you and your spouse/partner”, “mostly your spouse/partner”, “entirely your spouse/partner”, “mostly someone else (another member of your household or someone from outside your household”, and “a mixture of you (and/or your spouse/partner) and someone else”.	Mental wellbeing operationalised as Yes/no answer to the question “[Has] your (or at least one member of your household’s) mental health and well-being been affected by the pandemic and crisis)?”.	N = ca. 25,000; no further specification.	Frequency analysis
Tani et al., 2021 UK [68] Three waves: 2017/18, April & May 2020	To (1) document the potential damage to the financial security of working parents during the first wave of the COVID-19 pandemic in the UK; (2) explain the relationships between financial insecurity and the homecare of children and the mental well-being of working parents; and (3) explore the heterogeneity of these relationships across gender and economic status among working parents.	Time-use in hours per week on a) childcare or b) home-schooling with categories i) less than 1 hour, ii) 1–7 hours, iii) 7–20 hours and iv) 20+ hours per week.	GHQ-12^2^ Likert score	N = 15,665 individuals who work (employed or self-employed), with n = 6,795 working parents (employed or self-employed persons living with a child younger than 18) of which 57% are women.	Descriptive statistics; frequency analysis.
The Fawcett Society et al., 2020 [69] UK 15–21 April 2020	To highlight the differential experiences during the coronavirus crisis by social groups at the intersection between racialisation, racialised socio-economic disadvantage, gender, and disability.	Changes in time spent on childcare, housework, and informal care (respondents were asked whether they had spent more, less or about the same time caring for other adults during the pandemic compared to before).	Life satisfaction, happiness and anxiety measured on a 0–10 scale with higher numbers indicating higher outcomes (nor further specification); subjective evaluation whether one finds social isolation difficult to cope with (yes/no answer; no further specification).	N = 3,280 parents with at least one child aged 11 or under, people with low income (below the median), and BAME respondents, with n = 448 BAME women, n = 401 BAME men, and n = 1,308 white women; weighted data used for analyses.	Frequency analysis
Zoch, Bächmann & Vicari, 2020 [70]Click or tap here to enter text. Germany May–June 2020^11^	To examine short-term consequences for care-arrangements and resulting changes in wellbeing among parents affected by closures of schools and childcare facilities during the COVID-19 pandemic in Germany.	Care-arrangements based on a variable distinguishing whether care is provided (1) exclusively by the mother; (2) exclusively by the father; (3) by both parents, (4) by a mixed care-arrangement consisting of care combinations by parents, family members, relatives, or formal emergency care; or whether (4) the child is predominantly unsupervised.	Parental wellbeing assessed via overall life satisfaction measured as an answer to the question “All in all, how satisfied are you with your life at the moment?” and family life satisfaction “How satisfied are you with your family life/your work?”; answers to all questions were measured on an 11-point Likert scale, ranging from 0 = “completely unsatisfied” to 10 = “completely satisfied”.	N = 1,450 respondents including n = 897 mothers with a 14-year-old child, n = 229 educated parents and n = 324 parents of all educational levels with at least one child under 14 (analysis sample based on all subsamples, weighted data used in analyses).	Multinominal logistic regression models

*Notes*: ACEs = Adverse Childhood Experiences. BAME = Black, Asian, and minority ethnic. GB = Great Britain. GHQ-12 = 12-Item General Health Questionnaire. SOEP = German Socio-Economic Panel. SWB = Subjective wellbeing. UK = United Kingdom.

^1^ The GHQ-12 is an indicator for mental/ psychological distress that is based on 12 items/ questions about respondents’ depressive, anxiety symptoms, confidence and overall happiness. Each item has four response categories ranging from 0 = “less than usual”, 1 = “no more than usual”, 2 = “rather more than usual”, and 3 = “much more than usual”. Scores of each item are summed up and combined into a total GHQ Likert score indicating the level of mental/ psychological distress ranging from 0 (the least distressed) to 36 (the most distressed). All studies that use the GHQ-12 Likert Score as mental health measure use Understanding Society: UK Household Longitudinal Study (UKHLS) data.

^2^ Sample 4 (Spain working adults) among eight samples.

^3^ Study waves include (t1) 23 March– 10 May 2020, (t2): 20 April– 14 June and (t3): 3 June– 2 August 2020; responses from a retrospective questionnaire were used as pre-pandemic reference data.

^4^ Study waves include January/February 2020 or 2017/19 (pre-COVID), April (1^st^ lockdown), May (1^st^ lockdown), June (schools reopened), July (easing), September (easing), November 2020 (2^nd^ lockdown), January (3^rd^ lockdown) and March (schools reopened) 2021.

^5^ Includes pre-pandemic reference data from 2018 for unpaid care work and from 2019 for life satisfaction.

^6^ Countries include Czechia, Estonia, Finland, Germany, Italy, Portugal, Sweden and "other countries" incl. Austria, Belgium, France, Ireland, Luxembourg, Slovenia, Spain, Switzerland, and the UK.

^7^ Study waves include (t1) 23 March—10 May 2020, (t2) 20 April—14 June 2020, (t3) 3 June—2 August 2020, and (t4) 26 March—6 April 2021.

^8^ Study waves include (t0) February 2020, (t1) April 2020, (t2) September 2020, (t3) November 2020, and (t5) December 2020.

^9^ Countries include 25 OECD countries with 6 non-European countries.

^10^ Countries include GB, USA, Canada, The Philippines, and Kenya; only GB data were extracted.

^11^ Includes pre-pandemic reference data from various cohorts between 2010–2019.

The majority of included studies used a cross-sectional study design (n = 20), of which seven studies applied a quasi-pre-post design with retrospective questions to measure differences before and during the COVID-19 pandemic [22, 45, 46, 54, 55, 65, 67]. Longitudinal study designs were applied in seven studies, most of which used data from the Understanding Society: UK Household Longitudinal Study (UKHLS) [1, 49, 56, 62, 68]. An overview of the types of studies included in this scoping review is provided in Table 4.

**Table 4:** Overview of type of studies.

Reference	Research methods			Study design	Data source	Data collection	Sampling	
	Qualitative	Quantitative	Mixed methods				Convenience	Representative
*Peer-reviewed research articles*								
Ashencaen Crabtree et al., 2021 [44]			x	Cross-sectional	Original data	Online survey	x	
Balenzano et al., 2020 [45]		x		Cross-sectional, retrospective pre-post design	Original data	Online survey	x	
Bartolj et al., 2022 [46]		x		Cross-sectional, retrospective pre-post design	Original data	Online survey	x	
Beno, 2021 [47]	x			Semi-structured interview	Original data	Online interviews using WhatsApp/ Mobile Instant Messaging	x	
Cannito & Scavarda, 2020 [48]	x			Semi-structured interview, country-case study	Original data	Online interviews	x	
Cheng et al., 2021 [49]		x		Longitudinal, quasi-experimental	Understanding Society: UKHLS Wave 9 (2017–18) & COVID-19 module (April and May 2020 waves)	(Internet) panel data		x
Clemens et al., 2021 [50]		x		Cross-sectional	Original data	Online survey	x	
Czymara et al., 2021 [51]		x		Cross-sectional	Original data	Online survey	x	
Giurge et al., 2020 [52]		x		Cross-sectional	Original data	Online survey	x	
Hipp & Bünning, 2021 [53]		x		Longitudinal, quasi pre-post design	Original data	Online survey	x	
Ohlbrecht & Jellen, 2021 [54]		x		Cross-sectional, retrospective pre-post design with explorative questions	Original data	Online survey	x	
Xue & McMunn, 2021 [1]		x		Longitudinal	Understanding Society: UKHLS COVID-19 module (April and May 2020 waves)	Internet panel data		x
Yerkes et al., 2020 [55]		x		Cross-sectional, retrospective pre-post design	Longitudinal Internet Studies for the Social Sciences (LISS)	Internet panel data		x
Zhou & Kan, 2021 [56]		x		Longitudinal	Understanding Society: UKHLS Waves 9 (2017–18) and 10 (2018–19) & first eight waves of COVID-19 module	(Internet) panel data		x
*Grey literature*								
Bolis et al., 2020 [57]		x		Cross-sectional	Original data	Online survey	x	
Bujard et al., 2020 [58]		x		Cross-sectional	Mannheim Corona Study	Online survey		x
Chung et al., 2020 [59]		x		Cross-sectional	Original data	Online survey	x	
Close the Gap & Engender, 2021 [60]		x		Cross-sectional	Original data	Online survey	x	
Destatis, WZB & BiB, 2021 [61]		x		Cross-sectional	Mannheim Corona Study	Online survey		x
Etheridge & Spantig, 2020 [62]		x		Longitudinal	Understanding Society: UKHLS Wave 9 (2017–18) & COVID-19 module (April 2020 wave)	(Internet) panel data		x
Eurocarers/ IRCCS-INRCA, 2021 [63]		x		Cross-sectional	Original data	Online survey	x	
Eurofound, 2020 [22]		x		Cross-sectional, pre-post design	Original data	Online survey	x	
Hübgen et al., 2021 [64]		x		Longitudinal	Corona Alltag Survey	Online panel survey	x	
Illing et al., 2022 [65]		x		Cross-sectional, quasi pre-post design, event study design	Original data	Online survey		x
Kalaylıoğlu et al., 2020 [66]		x		Cross-sectional	Original data	Telephone-based survey (CATI)		x
OECD, 2021 [67]		x		Cross-sectional, quasi pre-post design	Risks that Matter (RTM) Survey	Online survey	x	
Tani et al., 2021 [68]		x		Cross-sectional	Understanding Society: UKHLS Wave 9 (2017–18) & COVID-19 module (April and May 2020 waves)	(Internet) panel data		x
The Fawcett Society et al., 2020 [69]		x		Cross-sectional	Original data	Panel data based on online survey		x
Zoch, Bächmann & Vicari, 2020 [70]		x		Longitudinal	National Educational Panel Study (NEPS-Corona_CAWI_C2 + various previous subsamples)	Panel data based on online survey		x

Notes: CATI = Computer-Assisted Telephone Interviewing. UKHLS = UK Household Longitudinal Study.

### COVID-19 containment measures

As shown in Table 3, data was largely collected at the early stages of the COVID-19 pandemic, as early as at the onset of the public health crisis in March 2020 when the WHO declared the Coronavirus outbreak a public health emergency and stay-at-home orders started the first lockdown in most European countries. Most studies were conducted between April and July 2020, during the phase of the first lockdown in which containment measures including shutdowns of schools, workplaces, and non-essential shops had been in force for a few weeks (April-May 2020), and during phases of reopening of schools (April-June 2020) as well as gradual ease of lockdown restrictions (May-July 2020). The studies administered by the OECD (2021) [67] and Close the Gap & Engender (2021) [60] were conducted during the phase of the second lockdown (October-December 2020) when restrictions were re-imposed.

While most surveys and interviews were conducted during lockdowns in general, some studies focused on more specific COVID-19 containment measures and related personal outcomes such as working from home as a result of workplace closures [44, 47, 48, 61, 64], adaptation of work hours or work patterns with regards to childcare and home-schooling duties [1, 53, 64], and general work-family conflict during lockdowns [59, 61, 68]. In the longitudinal study reported by Zhou and Kan (2021), participants were asked whether they tested positive for COVID-19 during each study wave to control for contracting the coronavirus so that the period indicator could better represent the spread of the Coronavirus and COVID-19 related policy change at the macro level. The cross-sectional studies by Hipp & Bünning (2021) [53] and Illing et al. (2022) [65] included multiple study waves which allowed for outcome parameters to be compared over different phases of national lockdowns, as did the longitudinal studies by Hübgen et al. (2021) [64] and Zhou & Kan (2021) [56]. The latter study used different study waves that coincided with different stages of COVID-19 policy responses in the UK over a one-year timespan (April 2020-March 2021).

### Measurements of unpaid care work

Table 3 depicts the definitions and operationalisation of unpaid care work underlying each study. Unpaid care work was mainly defined as housework (e.g., cleaning, preparing meals, day-to-day shopping) and personal care of household or family members within the nuclear family, consisting of mothers, fathers, (grand-)children and grandparents. Studies that focused on parents surveyed heterosexual (and predominantly coupled) parents. Only one study reported results for single parents [22] and two studies compared results of people with and without children in the household [58, 62]. The main measures were self-reported time spent on unpaid care work (n = 12) and the perception of equal sharing and primary caretaking between different-gender couples (n = 11). Seven studies provided subjective frequency data on the change in unpaid care work during lockdowns compared to the time before the COVID-19 outbreak. Examples of this were outlined in three qualitative studies (partly as part of a mixed-methods approach), in which changes in unpaid care work between women and men due to COVID-19 containment measures were examined in more detail [44, 47, 48].

Gender-specific differences in unpaid care work were mainly found for women in almost all studies. Ashencaen Crabtree et al. (2021) [44] and Balenzano et al. (2020) [45] found evidence of an unequal division of care work for women only. Although fathers were relatively more involvement in unpaid care work, mothers remained the primary caregivers and homemakers [45, 48, 55, 58, 64, 65]. While Chung et al. (2020) [59] found a tendency towards a more balanced distribution within couples as a result of the COVID-19 pandemic, Cannito & Scavarda (2020) [48] summarise that the division of unpaid care work did not change during lockdowns, especially for less egalitarian couples. Zoch et al. (2020) [70] emphasise that conforming to traditional gender roles seemed to increase the likelihood of an unequal care work division in the form of lower involvement of fathers and exclusive involvement of mothers in childcare.

Parenthood was identified as an important influencing factor in that gender differences in unpaid care work were greater for parents than for non-parents [52] or that parenthood was a more important predictor of reduced paid work during the pandemic than being a woman [53]. Being a single parent was associated with a greater childcare burden than being a coupled parent, especially being a single mother with children under 12 years of age [22].

Illing et al. (2022) [65] reported a higher childcare burden for parents of school-age children compared with parents of younger children. Zoch et al. (2020) [70] found that the share of fathers involved in childcare was greater for parents with younger children (under 14 years), while four other studies reported a greater gender care gap for parents of children aged 12 or younger at the expense of mothers [22, 64, 65, 67]. Parents of younger children also had greater difficulty reconciling paid work and unpaid work [44, 47].

The educational level of parents appeared to increase the likelihood of a more egalitarian division of additional care work. Before the COVID-19 pandemic, academic parents reported sharing childcare more equally than parents with lower education levels. During the pandemic, academic mothers reported being solely responsible for additional childcare tasks compared to academic fathers. Regardless of this, childcare during the pandemic was more evenly distributed among academic parents than among parents with lower levels of education [64]. Additionally, parents with a higher level of formal education reported having more access to formal emergency care in the first months of the pandemic and were more likely to rely on relatives for childcare [70].

In terms of parents’ employment status, being unemployed, working part-time and short-time were associated with taking on the majority of care work [53, 58, 64]. Although his was true for both women and men, working arrangements (i.e., working part-time vs. full-time) remained highly gendered [47, 53], and both working and non-working women spent more time doing housework than men [22]. According to a survey by the OECD (2021) [67], the gender care gap was largest in constellations where the mother was not working and the father was working. At the social policy level, the gender care gap in OECD countries was greater in countries where there were prolonged school closures and less was spent on family support (e.g., Türkiye, Poland and Ireland) and vice versa (e.g., Norway and Denmark).

Working from home was an important lockdown-induced change in working arrangements, and women were more likely to be working from home than men [61, 64]. As opposed to working on site, it was associated with an increase in unpaid care work for women and men [53]. In cases where fathers worked remotely, mothers were significantly more likely to become exclusive caregivers. In contrast, mothers’ status as essential workers, working longer hours or working on-site did not affect the likelihood of greater paternal or shared unpaid care work [70]. Hübgen et al. (2021) [64] saw a greater chance for equal housework division when both parents work from home.

Only one study reported findings for unpaid care work by ethnicity and showed that ethnic minority or racialised people performed more unpaid care work during compared to before the lockdown than white people [69]. According to this study, the gender gap was greater among white people; white men were the subgroup that performed the least unpaid amount of care work, while ethnic minority and racialised women performed the most.

While the average number of hours per week spent on informal care for sick, disabled or elderly persons increased by nearly a fifth during the lockdown, as European study shows [63] women caregivers spent more time on informal care. Lockdown-related restrictions on health and social services and other COVID-19-related difficulties led to an intensification of informal caregiving tasks, including emotional support, remote communication, practical help, care coordination and support, and help with transportation. In all these tasks, women caregivers reported a higher intensity than men caregivers [63]. The gender gap in informal caregiving was confirmed by two other included studies [62, 69]. One study also found that ethnic minority or racialised people, especially women, spent more time caring for adults, while white people were less likely to do so, with white men spending the least amount of time on informal care [69].

### Measurements of mental health

Definitions of mental health, wellbeing, quality of life, life satisfaction, and related concepts in health sciences vary widely in the included literature. These concepts and corresponding outcome measures are often intertwined (e.g., quality of life is a subjective aspect of mental health and wellbeing is an aspect of quality of life). Some measures relate to more objective levels of mental health, while others relate to subjective aspects of wellbeing. The most commonly used mental health measure in the included studies was satisfaction with various aspects of life (n = 11), such as satisfaction with work life and family life [48, 53, 58, 61] and general life satisfaction [54, 64, 65, 69], which was measured using predefined scales. The perceptions and frequency of (parenting) stress [45, 54], anxiety [60], emotional exhaustion [46], and more general depressive symptoms [48, 54, 57, 63, 64, 66, 69] were also frequently used. Five studies investigated subjective wellbeing [22, 47, 52, 59, 67]; the Eurofound (2020) [22] study used the 5-item World Health Organization Well-Being Index (WHO-5). A further five studies, all using UKHLS data, used psychological distress as their primary mental health outcome, measured with the 12-item General Health Questionnaire (GHQ) [1, 49, 56, 62, 68]. Czymara et al. (2021) [51] addressed the concept of mental load (i.e., concerns, worries, personal experiences) as a theoretically relevant dimension of gender inequality. They refer to cognitive labour, i.e. the work of anticipating needs and identifying options for meeting those needs, to illustrate gender inequalities in experiences and concerns during the lockdown. An overview of the definition of mental health adopted in the included studies is provided in in Table 3.

Most studies (n = 21) showed that it was mainly women’s mental health that deteriorated during the pandemic. Analyses of UKHLS panel data suggested a connection between the amount of time spent on unpaid care work and gender differences in mental health: Respondents who already had childcare responsibilities before the pandemic showed lower levels of wellbeing during the pandemic [62]. The deterioration in wellbeing was significant for parents with substantial childcare responsibilities (i.e. over 15 hours a week), which applied to 70% of the surveyed mothers. An increase in weekly housework hours for women also appeared to be associated with a deterioration in their wellbeing. The wellbeing levels of men, who took on a much smaller share of housework already, appeared to be unaffected. The increase in informal care for people outside the household appeared to affect the mental health of women and men carers equally, despite the gender gap in informal caregiving (51% women vs. 45% men).

Tani et al. (2021) [68] came to similar conclusions: an intensive amount of time spent on childcare and home-schooling of at least 20 hours per week was associated with a deterioration in mental health. Working mothers and parents whose pre-pandemic income was above the median were particularly affected. The authors conclude that working mothers (vs. working fathers) and working parents of lower income households (vs. working parents of higher income households) had a poorer mental health and financial situation during the pandemic. Financial insecurity was identified as a predictor of poorer mental health for mothers and fathers in two other studies [49, 62]. A privileged living and working environment (i.e., more living space, remote working opportunity, family support) also seemed to make it easier for Austrian parents to reconcile working from home and home-schooling during the lockdown [46].

Generally speaking, persons with childcare responsibilities seemed to be more severely affected by lockdown-related mental health deterioration than non-parents [46, 53, 59, 62]. In contrast, results from the Mannheim Corona Study [58] suggested that women and men without children in their household were particularly dissatisfied with their family life as a result of the contact restrictions in Germany.

The age or schooling stage of children seemed to make a difference in how parents were coping with lockdown-induced additional caregiving responsibilities [47, 55, 58, 65]. Parents of younger children more frequently reported difficulties in reconciling work and family life [47]. For example, German mothers with children over the age of 6 were particularly dissatisfied with their work and family life [58]. The life satisfaction of German fathers, on the other hand, did not appear to be influenced by the age of their children [65]. Yerkes et al. (2020) [55] found significant gender differences in the quality of life among Dutch parents: Mothers reported a greater loss in their leisure time compared to father (57% vs. 36%); the presence of children of primary school age was a significant explanatory factor. A deterioration in work-life balance and a significant increase in disagreements about childcare division during the lockdown was observed for parents regardless of gender. Having primary school children appeared to be a more relevant explanatory factor.

Caregivers’ working arrangements seemed to have had an influence on parental wellbeing. For example, mothers who worked from home reported to be more satisfied with their work and family life than parents and non-parents who had to work on-site [58, 61]. Moreover, unpaid caregivers in essential work were more likely to report worse mental health outcomes than non-essential workers [55, 57].

They way in which parents perceived changes to their work and family life caused by the lockdown appeared to be gendered. The qualitative accounts of Italian parents reported by Cannito & Scavarda (2020) [48] showed that fathers believed that family interfered with work, leading to reduced productivity, feelings of guilt toward the workplace and higher stress levels. For the interviewed mothers, on the other hand, work interfered with family life, preventing them from responding to their children’s needs and from mentally detaching themselves from their work, accompanied by feelings of guilt toward their children. Similarly, British mothers were more likely than fathers to report that their work prevented them from spending time with their family (48% vs. 40%) and that their family prevented them from spending time with their work (49% vs. 32%) [59]. Czymara et al. (2021) [51] summarise that these differences in concerns as one part of cognitive labour reflected the social situation of women and men, namely one in which the traditional division of paid and unpaid work follows the male breadwinner model, even among the relatively well-educated.

Ethnic minority and racialised people appear to be disproportionately affected by COVID-19 containment measures, not only in terms of a greater caregiving burden, as previously mentioned, but also in terms greater deterioration in mental health. In Scotland, mothers were more likely to report higher anxiety levels than fathers; ethnic minority and racialised parents, especially mothers, reported the highest anxiety levels [60]. In line with these findings, another study from the UK shows although there were no statistical differences in coping levels with social isolation at the intersection of gender and ethnicity/racialisation, scores for life satisfaction and happiness were lowest for women of colour and highest for white men. Consistently, anxiety levels were highest for women of colour and lowest for all men. Overall, white men were most likely to have high levels of life satisfaction and happiness and, along with men of colour, the least likely to have high anxiety scores. All women were equally least likely to have high life satisfaction and happiness scores and high anxiety scores [69]. One study reported anxiety levels by disability status and found that disabled mothers were most likely to report high anxiety levels (58%) compared to non-disabled mothers (32%) and non-disabled fathers (24%) [60]. However, the authors did not provide data on unpaid care work by disability status. A summary of the findings on gender differences in unpaid care work and mental health can be found in Table 5.

**Table 5:** Gender and intersectional differences in unpaid care work and mental health.

Author(s), year	Observed gender differences in unpaid care work	Observed gender differences in mental health	Observed mediators/ predictors	Observed intersectional differences
*Peer-reviewed research articles*				
Ashencaen Crabtree et al., 2021 [44]	For women only	Mainly for women	None reported	None reported
Balenzano et al., 2020 [45]	For women only	Marginal differences	None reported	None reported
Bartolj et al., 2022 [46]	Mainly for women	Marginal differences	None reported	None reported
Beno, 2021 [47]	Mainly for women	No differences	Work-childcare compatibility was expressed as especially difficult by parents of younger children.	
Cannito & Scavarda, 2020 [48]	Mainly for women	Mainly for women	None reported	None reported
Cheng et al., 2021 [49]	Mainly for women	Marginal differences	Working parents with pre-pandemic income above the median show the strongest relationship between child-related activities and mental health.	None reported
Clemens et al., 2021 [50]	Mainly for women	Mainly for women	None reported	None reported
Czymara et al., 2021 [51]	Mainly for women	Mainly for women	None reported	None reported
Giurge et al., 2020 [52]	Mainly for women	Marginal differences	Parental status as moderator for time-use on necessities and work, such that gender differences were larger in parents vs. non-parents.	None reported
Hipp & Bünning, 2021 [53]	Marginal differences	Mainly for women	Parenthood (for working patterns) as more important predictor than being a woman for reduced working hours or not to be working at all during the pandemic; being the only partner in paid work & being the only partner to work onsite vs. working from home (for unequal unpaid work division).	None reported
Ohlbrecht & Jellen, 2021 [54]	Mainly for women	Mainly for women	Educational attainment (for aspects of life satisfaction; not reported by gender).	None reported
Xue & McMunn, 2021 [1]	Mainly for women	For women only	Partnership moderates the association between adapting work patterns and the GHQ-12 Likert score among women.	None reported
Yerkes et al., 2020 [55]	Mainly for women	Mainly for women	Being an essential-worker parent (for reduced leisure time/ quality of life); having children in primary school (for differences in leisure time & disagreements about division of childcare tasks); parental educational level and schooling stage of children (for differences in perceived work-life balance).	None reported
Zhou & Kan, 2021 [56]	Marginal differences	Marginal differences	Being a member of BAME population group & being a keyworker (for lockdown-impact on earnings reduction).	None reported
*Grey literature*				
Bolis et al., 2020 [57]	Mainly for women	Mainly for women	Being a key worker (for mental wellbeing impairments).	None reported
Bujard et al., 2020 [58]	Mainly for women	Mainly for women	The presence of children in household (yes/no), age group of children (under/over 6 years), type of work arrangement (working from home, working onsite, short-time work) of oneself and of one’s partner for the for time spent on unpaid care work and levels of satisfaction.	None reported
Chung et al., 2020 [59]	Mainly for women	Mainly for women	Being a parent (for higher likelihood of negative feelings, and lower likelihood of positive feelings).	None reported
Close the Gap & Engender, 2021 [60]	Mainly for women	Mainly for women	None reported	Compared with 28% percent of white mothers and fathers, 34% of BME mothers and 32% of BME fathers reported high levels of anxiety. Compared with non-disabled mothers (32%) and non-disabled fathers (24%), 58% of disabled mothers reported a high level of anxiety
Destatis, WZB & BiB, 2021 [61]	Mainly for women	Mainly for women	Type of work arrangement (working from home vs. working onsite) for time spent on unpaid care work and levels of satisfaction.	None reported
Etheridge & Spantig, 2020 [62]	Mainly for women	Mainly for women	There is a stronger decline in wellbeing for those who report a worse subjective financial situation (women and men do not seem to be differently affected with regards to financial measures); different levels of loneliness are differently related to changes in wellbeing: for those reporting being lonely, the correlation between loneliness and changes in wellbeing is strongly increasing in reported loneliness, while true for both gender groups, the fraction of women affected is higher; those who report less loneliness show substantially higher wellbeing, while those who report an increase in loneliness are substantially less happy; individuals with more close friends face larger declines in wellbeing, true for both gender groups, but again women are slightly disproportionally affected; demographics: being in a couple seems to have only comparatively benefitted men; those with children face slightly larger declines in wellbeing than those without; youths (16–30) of both gender groups face substantially larger decline in wellbeing than older individuals.	None reported
Eurocarers/ IRCCS-INRCA, 2021 [63]	Mainly for women	Mainly for women	None reported	None reported
Eurofound, 2020 [22]	Mainly for women	Mainly for women	None reported	None reported
Hübgen et al., 2021 [64]	Mainly for women	Mainly for women	Educational attainment (having a university degree vs. medium or lower educational attainment) and being employed/working on-site (vs. WFH/being unemployed) in terms of more equal care work division.	None reported
Illing et al., 2022 [65]	Mainly for women	Mainly for women	Age category of children (for time-use on childcare and level of life satisfaction).	None reported
Kalaylıoğlu et al., 2020 [66]	Mainly for women	Marginal differences	None reported	None reported
OECD, 2021 [67]	Mainly for women	Mainly for women	Employment status of oneself/ one’s partner (for the likelihood of taking on additional unpaid care work).	None reported
Tani et al., 2021 [68]	Mainly for women	Mainly for women	Financial insecurity (for worse mental health), household income (for worse mental health).	None reported
The Fawcett Society et al., 2020 [69]	Mainly for women	Mainly for women	None reported	BAME (Black, Asian and Minority Ethnicity) people perform more unpaid care work relative to pre-pandemic situations and relative to white people (except for white women who perform less than BAME women, but more than BAME men; least performed by white men).
Zoch, Bächmann & Vicari, 2020 [70]	Mainly for women	Marginal differences	Age group of children (younger than/ aged 14), educational level of parents, working conditions of parents (working from home/onsite), changes in work time (same/increased/decreased), gender role attitudes, key-worker status (for differences in care arrangements in families).	None reported

Notes: BAME = Black, Asian, minority ethnic. BME = Black and minority ethnic. GHQ-12 = 12-Item General Health Questionnaire.

### Concepts of gender and intersectionality

To account for the degree of their inclusion, we extracted data on the conceptualisation of gender, gender (in-)equality and mention of intersectionality (see S4 Table). The gender or biological sex of respondents was measured as a binary variable in all studies, with the exception of two studies in which it was measured as a categorical variable (female, male, diverse) but reported as a binary variable due to the small number of diverse individuals in the samples [51, 54]. Some studies referred to theories and theoretical concepts to support their arguments and hypotheses, including neoliberalism and the ideal worker notion [44], role theory [47], different fatherhood models, the concepts of femininity and masculinity [48], and ‘doing gender’ [51, 53]. Other authors discussed gender inequalities in the context of socio-cultural narratives and geographical contexts, highlighting traditionalist or conservative societal norms that promoted gender gaps in employment and unpaid care work in Italy [45, 48], Germany [53], the Netherlands [55], the UK [56], and Türkiye [66]. Two studies collected data on attitudes towards gender roles and found that more traditional gender role attitudes were associated with lower levels of fathers’ childcare involvement [70]. Hübgen et al. [64] found that women in Germany appeared to have more progressive views towards gender roles than men; the same was true for university graduates compared to non-academics.

The theoretical concept of intersectionality was explicitly mentioned in two reports [60, 69], which emphasised the importance of including gender, racialisation, disability and other lived realities in the analyses of COVID-19 related mental health and social disadvantage of racialised and disabled women. Nine other publications implicitly integrated intersectional approaches into their gender analyses by including other dimensions of social inequality such as socio-economic status [45, 52], working arrangements and employment situation [53, 58, 61, 70], parenthood [53], education [54, 64, 70], and financial situation [68].

## Discussion

### Summary of results

The aim of this scoping review was to explore the impact of European policy measures to contain the spread of COVID-19 on the division of unpaid care work and related changes in mental health between gender groups, with a focus on intersectional dimensions of potential gender inequalities. As expected, the collection of evidence published up to two years after the outbreak of the pandemic largely confirms that women bore the greatest burden of the COVID-19 crisis, as predicted by empirical work before [7–10] and during the pandemic [22]. The time spent on childcare, housework and informal caregiving was already gendered before the pandemic, with women taking on these traditional caregiving responsibilities more often than men [2, 3]. Although men’s unpaid care work appears to have increased during lockdown phases, especially among different-gender coupled parents, it did not reach women’s degree of involvement. What is more, women caregiver’s mental health was the most affected during the pandemic when compared to men caregivers, largely due to greater involvement of women in unpaid care work. Although a trend toward a more egalitarian division of unpaid care work can be observed in European countries, the gender care gap remains.

### Intersections of parenthood, paid work, and gender

Parenthood has proven to be one of the biggest influencing factors on caregivers’ mental health during the lockdown. Parents, most notably mothers and working parents, showed a greater overall deterioration in mental health than non-parents [52]. Parents of school-age children seemed to be particularly affected by school closures and home-schooling efforts [44, 47, 65]. Balancing work and family life during the pandemic appeared to be a particular challenge for working parents. This was strongly linked to the amount of time spent on childcare and home-schooling [49, 62, 68] as well as financial insecurity [49, 68]. Lockdown-induced work and care arrangements were highly gendered [47, 53] and both working and non-working women spent more time on unpaid care work than men [22, 67]. Working mothers had a higher overall burden from the combination of paid work, childcare and education, housework and commuting [65] and showed poorer mental health outcomes than working fathers [49, 55, 68].

### Intersections of working arrangements and gender

Working constellations affected how caregiving was distributed between partners: the gender care gap appeared to be largest in the most traditional work-care arrangements, where women were not employed and men were employed [67]. However, the results are inconclusive: Analyses from the initial phase of the lockdown in Germany (March and April 2020) showed that in constellations in which only one partner was employed or working onsite, this partner performed less unpaid care work than their non-employed or remote-working partner, regardless of gender [53]. A few months into the German lockdown, in May and June 2020, fathers’ remote work contributed significantly to exclusive maternal caregiving, while mothers’ working constellations did not promote paternal or shared caregiving [70]. Although working from home increased caregiving for both gender groups [53], women were more likely to work remotely than on-site compared to men [61, 64]. Parents working in essential sectors (e.g., healthcare, social services, day care, education, supermarkets) were more likely to report poorer mental wellbeing during the COVID-19 pandemic than non-essential workers [55, 57], especially if their children were attending primary school [55]. At the same time, women were more likely to work in essential sectors during the pandemic and took on the majority of unpaid care work. While the COVID-19 pandemic brought challenges, it also provided working parents with opportunities to experience positive changes in their daily routines and family dynamics. Remote work allowed some parents more flexible working arrangements and a better work-family balance, especially for mothers [48, 58, 61]. This required the possibility of working remotely and prerequisites that contributed to a privileged and less stressful working-from-home environment, such as a spacious apartment with a separate room [47]. Moreover, remote-working parents had more opportunities to spend time with their children, which appeared to be beneficial for mothers’ wellbeing [44].

### Intersections of financial security and gender

Financial insecurity has proven to be an important predictor of poorer mental health for both mothers and fathers [49, 62], with working mothers and working parents of poorer households experiencing higher levels of mental and financial distress [68]. Working women suffered less earnings reduction than working men during the COVID-19 pandemic, largely due to the higher proportion of women working in essential fields such as health and social care. Nevertheless, women’s wellbeing and mental health were more sensitive to COVID-19 containment measures compared to men, with greater gender differences during the more restrictive phases of lockdowns [56].

### Intersections of racialisation/ethnicity, disability, and gender

The results of this scoping review highlight that, among European women, population groups that are often understudied, such as women who are single parents, disabled or of colour, have the greatest increase in unpaid care work and the greatest deterioration in wellbeing [22, 60, 69]. The lack of diversity in study populations is a short-coming of the studies included in this scoping review. All included studies that reported results at the intersection of gender and racialisation, ethnicity or migration status used the umbrella terms ‘BAME’ (black, Asian and minority ethnic) and ‘BME’ (black and minority ethnic). This is highly critical as it emphasises some groups to the exclusion of others and portrays people of colour as a homogenous group, leaving no room for differences between and within different migrant and racialised groups. Similarly, disabled unpaid caregivers had much higher levels of anxiety than able-bodied parents [60], however all but one study failed to consider a disability perspective in their analyses altogether. In terms of intersecting social dimensions and lived realities, researchers need to consider more diverse study populations and more innovative ways to reach them, e.g., through qualitative interviews and participatory approaches.

### Intersections of societal norms and gender

The division of unpaid care work between gender groups, especially in different-gender coupled households, is not only the result of negotiation processes between gender groups (especially between romantic partners), but it is also strongly influenced by the underlying societal norms that shape the roles of women and men in society and the expectations placed on them in terms of paid and unpaid labour [4, 5]. As the uptake and division of unpaid care work between women and men is highly gendered and normalised, measures to contain COVID-19 would naturally affect gender groups differently, as women and men are likely to adjust their behaviour to what is expected of them based on gender-normative ideas [71].

Most of the included studies lacked theoretical approaches to analyse the underlying mechanisms of gender inequality. These include most prominently ‘doing gender’ [71], bargaining position [72] and time availability, as highlighted by Steinmetz et al. (2022) [73], which are all necessary to understand gender inequalities during COVID-19 (see S4 Table). For example, changes in time availability affect the bargaining position in couple households [73] in that less time for commuting and school trips means more time for childcare and housework for parents, especially for fathers. Adherence to gender-normative beliefs, according to which women and men adapt their behaviour to societal expectations, would naturally lead to a greater involvement of mothers in unpaid care work and less or unchanged involvement by fathers [70]. Contrary to initial debates, we find no evidence of a re-traditionalisation of gender roles, whereby women, especially mothers, would shoulder the additional unpaid care work induced by the various containment measures [74]. However, we cannot conclude that the COVID-19 pandemic promoted a more egalitarian division of unpaid care work either, although we do see a trend towards greater involvement of men and fathers in personal care and housework [59].

This is particularly evident in more conservative welfare states with more traditional gender roles, such as in Italy and Türkiye, where women took on additional care work, especially childcare, when external care services were not or were no longer available, as has been the case during the COVID-19 pandemic [67]. This conservatism in European countries is reflected in the gender care gap, with a more traditional division of unpaid care work especially in Italy [45, 48], Germany [50, 51, 58, 70], Austria [47], and the Netherlands [55]. In some liberal welfare states such as the UK, however, there is a trend towards a more egalitarian division of paid and unpaid labour between women and men [59]. This is confirmed by studies in other liberal welfare states, such as the United States [75], Canada [76] and Australia [77].

The changing demands on unpaid care work also translate into changes to working arrangements [70]. In terms of working conditions, more working women than working men reduced their working hours or even quit their jobs to care for their children during the pandemic, although the male-breadwinner and female-caretaker models do not seem to be holding [53]. The reduction in working hours suggests that the ‘one-and-a-half-earner’ model [78] appeared to prevail in European countries and was being reinforced during the pandemic. This favoured a more traditional division of unpaid care work, even if this is not true for all people and population groups studied.

### Scope and limitations

Despite rigorous research practices underpinning this scoping review, there are some limitations to our study. Firstly, while our goal was to include a wide range of studies to capture different perspectives, we acknowledge the potential for selection bias, as studies from high-income Western European countries predominate in the final selection and other parts such as Eastern Europe were excluded. The gender-specific division of paid and unpaid labour is embedded in the specific socio-economic and socio-political structures of a society and fundamentally determined by these circumstances. This also applies to mental health, which is influenced by interactions between individual and socio-structural conditions. The relationship between gender, unpaid care work and mental health, on which this review is based, is situated in a specific socio-economic and socio-political context, namely in high-income Western European countries with predominantly liberal and conservative welfare states. Therefore, the generalisability of our results to other societal and structural contexts is limited. This limitation reflects the availability of research on this topic. Since gender inequalities were found even in these contexts, we assume that these differences are more pronounced in other economies and more conservative welfare states. Nonetheless, the literature searches were conducted in English-language sources using English search terms, which may have led to an under-representation of non-English literature that could have contributed to a more diverse set of findings.

Secondly, although we tried to include a range of study designs, the predominance of quantitative studies in the final selection could introduce a bias in favour of certain research methods. Future studies could employ more diverse study designs, especially qualitative ones, to mitigate this limitation. Most studies were based on online surveys, which require access to digital devices and a certain level of digital literacy to participate. While online surveys were essential for data collection during the COVID-19 pandemic, there is a risk of over-representation of people with better access to digital devices and higher levels of digital literacy. This could affect the generalisability of the results to populations with limited internet access and digital literacy.

Thirdly, a limitation of scoping reviews is the neglect of quality assessment of included studies. The studies included in this review differ in terms of study populations, cultural contexts of countries, pandemic responses and the type of data used. In the early stages of the COVID-19 pandemic, ad hoc data of varying quality was collected, ranging from highly selective convenience samples to probability-based longitudinal data (see Table 4). Most studies lack pre-pandemic information, while some use retrospective measures with a potential recall bias [79]. Only a few studies investigated the impact of specific COVID-19 containment measures or included them as measurable instruments in their analyses. These include longitudinal studies with multiple study waves that correlate with different lockdown phases. The most recent survey in this scoping review refers to spring 2021, meaning that our study covers the short-term impacts of COVID-19 containment measures on unpaid care work and mental health. To investigate longer-term impacts and causal effects, more recent studies and further longitudinal studies with elaborate methodology are needed.

Fourthly, as noted in some studies, the populations under study consisted of highly educated and more financially stable individuals, which means our results are biased towards middle- or higher-class populations. At the same time, racialised individuals, migrants and ethnic minorities, as well as working-class individuals and those in precarious or irregulated working arrangements are heavily underrepresented, further limiting the generalisability of our results.

What is more, conducting a scoping review on a phenomenon such as the COVID-19 pandemic is subject to limitations and challenges given how rapidly it is changing. This scoping review mainly included cross-sectional studies, which are not able to depict changes in outcome parameters over time. Therefore, most studies were not able to compare different pandemic periods and related unpaid care work and mental health outcomes. The longer-term impacts and effects on the consequences of the gendered division of unpaid care work on the mental health of caregiving women, men and gender-diverse persons remain unclear. We expect further studies on this topic in the upcoming years. So far, we have been able to identify basic patterns of how COVID-19-related containment measures affect unpaid care work and mental health differently. We were also able to identify potentially at-risk-groups for increased caregiving burden and worsening mental health.

Finally, given the wide range of measures of unpaid care work and mental health included in this scoping review, the results must be interpreted with caution. There are a variety of measures of unpaid care work, with few studies using measures of time-use and others relying on subjective relational and retrospective measures, which are more prone to bias. Additionally, self-reported measures of unpaid care work may underestimate women’s share of caregiving within couple relationships, while men tend to overestimate their actual share of childcare and housework [80]. There is an even greater variety of measures in mental health, ranging from screening instruments for psychological distress and overall mental health to measures of wellbeing and quality of life. Arguably, measures of wellbeing, satisfaction and quality of life are not measures of mental health, but rather mediators that help explain how different phases of COVID-19 lockdowns affect mental health, or moderators that influence the magnitude or direction of the impact on mental health [81]. Although not necessarily equivalent to direct measures of mental health disorders, measures of wellbeing and quality of life provide valuable insights into individuals’ daily lives and challenges [82]. Our aim was to include as many studies as possible using a broad definition of mental health. Future studies should use multiple types of caregiving and mental health measures to obtain a more nuanced picture.

### Gaps and future directions

Unpaid care work remains largely invisible, not only in traditional families, but also in academia. In many studies on the impact of COVID-19 containment measures on mental health, unpaid care work was not measured, and when included, many authors did not report their results by gender. This is reflected in the number of full-text articles (n = 43) we had to exclude (see Fig 1). Surprisingly, informal caregiving to elderly, sick and disabled people was the least gender-disaggregated form of unpaid care work, although informal caregivers make up 10–25% of the European population and are predominately women [83].

None of the included studies considered non-heterosexual individuals, same-sex couples and non-heteronormative constellations of families and unpaid care work, which was often due to the far too small number of non-heterosexual or non-cis-gendered individuals in the various samples. Investigating the distribution of unpaid care work in queer couples and differences in mental health between partners, for example, could shed light on negotiations in partnerships and households where heteronormative beliefs may have less influence. Evidence from before the COVID-19 pandemic suggests that same-sex couples have a more egalitarian distribution of housework compared to opposite-sex couples, with gender-affirming behaviour being less important and the availability of time and relative economic resources being more taken into account [84, 85].

Future research and policy must broaden their focus from the ‘typical’ unpaid care work and its ‘traditional’ distribution between women and men, mothers and fathers, to the cognitive and emotional dimensions of gender inequality. Unpaid care work consists not only of practical activities, but often also of cognitive and psychological processes, such as organising, planning and managing family work, worrying and anticipating needs. These types of cognitive and emotional labour have far more stressful impacts on caregivers and may lead to greater gender disparities in mental health [51, 86], but are largely overlooked in the literature on gender inequalities. Our findings suggest that cognitive and emotional labour was gendered and resulted in higher mental load for women, particularly mothers, as evidenced by higher parental stress levels [59, 68], worries related to children’s wellbeing [48] and greater dissatisfaction with unpaid care work division [45, 50] compared to men and fathers.

Another factor that should be taken account in future studies is the contextualisation of results with regard to other policies (e.g., family, equality, and labour-market policies) and the socio-political characteristics of individual European countries. Some of the studied countries reacted very differently to the pandemic, which does not make it easy to compare different settings. Future studies need to address unpaid care work, gender equality and diversity, as well as policies to promote them. Measures to combat the pandemic also varied greatly and need to be analysed in terms of gender inequality and other disadvantages, as well as policies that address them.

## Conclusion

The outbreak of the COVID-19 pandemic within Europe and, in particular, the introduction of policy measures to contain the spread of the coronavirus have changed social and economic life. This is also the case worldwide, leaving everyone affected. This includes people with unpaid caregiving obligations being forced to adapt to new circumstances in their daily lives and re-negotiate their roles in their families and care communities. The closure of schools, day-care centres and social-care facilities, as well as the loss of social support from family members and friends, led to an increase in childcare and personal care duties, especially for parents of younger children. Changes in working arrangements, such as reduced working hours and remote work, brought new challenges for the work-life balance and financial situations of caregivers. Caregivers in essential work were particularly challenged as they had fewer or no options for outsourcing additional childcare and home-schooling demands.

The COVID-19 pandemic is over—at least the last containment measures have been lifted, even in the most restrictive countries. However, the end of the lockdown does not mean the end of the struggle for caregivers; they must now return to their ‘normal’ daily lives and cope alone with the mental strain and work-family conflicts that the protracted COVID-19 pandemic brings. Does the end of the COVID-19 pandemic and related containment measures also mean an end to research on the socio-economic and socio-political consequences of the pandemic? Now is the time to learn from the consequences of the pandemic for different population groups, which calls for increased research in this area. Of course, there is also the question of how family life and the distribution of care work has changed in Europe, especially in different-sex or, more precisely, heteronormative partnerships, which seem to adhere to traditional gender roles despite the pandemic.

To summarise, this scoping review provides some new insights to the gendered impact of COVID-19 containment measures on unpaid care work and mental health in Europe. Although our study is limited to a few but well-established European economies, our findings suggest a wider gender gap in the division of unpaid care work and, to a lesser extent, mental health, which is unfavourable for women and mothers. Nevertheless, we see a break in the traditional division of childcare, with fathers taking on a greater role in family work, which makes us optimistic about the division of unpaid care work in the post-COVID-19 era.

Given the gaps identified in the scientific literature, there is a need for research initiatives on the social and health impacts of unpaid care work as a highly gendered and essential pillar of social welfare. Research collaborations are needed between high- and low-income countries and between different types of welfare states to share knowledge and learn from each other. To achieve better representation of at-risk and often overlooked groups in quantitative and qualitative studies, community-based and participatory approaches should be integrated. Participatory approaches can help shed light on the experiences of at-risk and marginalised population groups with different care arrangements and lived realities in dealing with stress and mental health problems in the post-COVID-19 era.

To address the issue of mental health and unpaid care work, future health policies need to consider the socio-economic diversities in our communities. Our findings show that despite the slightly reduced gender care gap compared to pre-pandemic levels, women were particular burdened by increased unpaid care work. Those who take on personal care responsibilities, such as parents and informal caregivers, need to be supported, especially women of colour and less socio-economically privileged women. To fully prepare for future pandemics, European health policies should actively seek to reduce gender inequalities in the division of paid work and unpaid care work. This should include diverse communities and consider factors that promote mental health related to caregiving burden.

## Supporting information

S1 Table Prisma SCR 2018 checklist. (PDF)

S2 Table Example search strategy for MEDLINE database. (PDF)

S3 Table Results of included research articles and grey literature including limitations. (PDF)

S4 Table Inclusion of gender and intersectionality. (PDF)

S1 Appendix Documentation of the grey literature search. (PDF)
