# Correction: Caring is not always sharing: A scoping review exploring how COVID-19 containment measures have impacted unpaid care work and mental health among women and men in Europe

**DOI:** 10.1371/journal.pone.0347221

**Published:** 2026-04-14

**Authors:** Hande Gencer, Regina Brunnett, Tobias Staiger, Hürrem Tezcan-Güntekin, Kathleen Pöge

The first author, Hande Gencer, is incorrectly noted as the senior author. The correct senior author is Kathleen Pöge.
