# Correction: Caring is not always sharing: A scoping review exploring how COVID-19 containment measures have impacted unpaid care work and mental health among women and men in Europe

**DOI:** 10.1371/journal.pone.0351654

**Published:** 2026-06-11

**Authors:** Hande Gencer, Regina Brunnett, Tobias Staiger, Hürrem Tezcan-Güntekin, Kathleen Pöge

There are errors in the Author Contributions. The contributions should indicate that authors Regina Brunnett, Tobias Staiger, Hürrem Tezcan-Güntekin contributed equally to this work.
