# Supplementary material for: Caring is not always sharing: A scoping review exploring how COVID-19 containment measures have impacted unpaid care work and mental health among women and men in Europe
Source: PLoS One. 2024 Aug 30;19(8):e0308381. doi: 10.1371/journal.pone.0308381 (PMC11364293; doi:10.1371/journal.pone.0308381)
Supplement: S2 Table — (PDF) [file pone.0308381.s002.pdf]

Caring is not always sharing: A scoping review exploring how COVID-19 containment measures have impacted unpaid care work and mental health among women and men in Europe

S3 Table: Example search strategy for MEDLINE database

**MEDLINE (via OvidSP), 01/03/2020-07/09/2022 (searched/exported: 07/09/2022)**
1. exp caregivers/
2. exp work-life balance/
3. 1 or 2
4. ((informal OR unpaid OR family OR familial OR spous*) adj3 (care or carer* or caregiver* or caregiving or care-work or "care work" or "care giver*" or care-giver* or care-giving or "care giving")).ti,ab.
5. ((unpaid OR unwaged OR domestic OR reproductive OR family OR familial) adj3 (work or worker* or labor or labour or laborer* or labourer*)).ti,ab.
6. (childcare or "child care" or child-care or elder-care or "elder care" or housework or household or work-life-balance or "work-life balance" or work-family-conflict or "work-family conflict" or work-to-family-conflict or "work-to-family conflict" or "family nursing" or "family-centered nursing" or "family centered nursing").ti,ab.
7. or/3-6
8. exp coronavirus/
9. exp sars-cov-2/
10. exp covid-19/
11. or/8-10
12. (covid OR covid-19 OR "covid 19" OR coronavirus* OR corona-virus* OR "corona virus*" OR 2019-nCov OR "2019 nCov" OR sars-cov-2 OR "sars cov 2" OR pandemic OR "Severe Acute Respiratory Syndrome Coronavirus 2").ti,ab.
13. 11 or 12
14. (lockdown* OR lock-down* OR "lock down" OR shutdown* OR shut-down* OR "shut down*" OR quarantine* OR "containment measure*" OR "shelter-in-place order*" OR "stay-at-home order*").ti,ab.
15. 13 and 14
16. 7 and 15
17. limit 16 to yr="2020 -Current"
18. exp mental health/
19. exp mental disorders/
20. exp psychological distress/
21. exp stress, psychological/
22. exp anxiety/
23. exp anxiety disorders/
24. exp caregiver burden/
25. or/18-24
26. ("mental health" OR "mental disorder*" OR "psychological distress" OR "psychological stress" OR anxiety OR "anxiety disorder*" OR "caregiver burden" OR "psychological burnout" OR burnout OR "mental wellbeing" OR "mental stability" OR "mental balance" OR "mental health problem*" OR "emotional suffering" OR burden OR exhaustion OR stress OR "psychosocial risk factor" OR "psychosocial impact" OR "psychosocial problem" OR wellbeing OR well-being OR "life satisfaction" OR "quality of life" OR depression OR depressive OR psychosocial OR psychological OR mental OR emotional).ti,ab.
27. 25 or 26
28. 16 and 27
29. limit 28 to yr="2020 -Current"
