# Supplementary material for: Caring is not always sharing: A scoping review exploring how COVID-19 containment measures have impacted unpaid care work and mental health among women and men in Europe
Source: PLoS One. 2024 Aug 30;19(8):e0308381. doi: 10.1371/journal.pone.0308381 (PMC11364293; doi:10.1371/journal.pone.0308381)
Supplement: S3 Table — (PDF) [file pone.0308381.s003.pdf]

Caring is not always sharing: A scoping review exploring how COVID-19 containment measures have impacted unpaid care work and mental health among women and men in Europe

S4 Table. Results of included research articles and grey literature including limitations

**Reference**	**Summary of key findings**
Peer-reviewed research articles	
**Ashencaen Crabtree et al., 2021**	Both the quantitative and qualitative data show clear gender differences in the way the lockdown was experienced with work-life balance: Descriptive analysis shows that work-life balance ‘’got worse‘‘ for most female academics (63.2% versus 45% of men; Chi-squared test showed no significance), while it ‘’improved‘‘ for 33.3% and ‘’remained unchanged‘‘ for 2.6%; for male academics, half “‘got worse‘‘ and half “”improved‘‘ (44.8% each) and for 10.3% it “”remained unchanged‘’. A Chi-squared test revealed significant gender differences in research activity, with women predominantly reporting a decrease (47% compared to 31% men) and men reporting an increase (48% compared to 16% women). Overall, the women's qualitative comments showed clear gender differences in terms of their perceived experiences: (a) huge increase in workload associated with online teaching methods since lockdown with an erosion of women's personal time; trying to maintain ‘business as usual’ behaviours during lockdown was perceived as very stressful; b) all respondents who reported a deterioration in work-life balance due to childcare, home-schooling and other caring responsibilities were women; regardless of keyworker status, female respondents appeared to struggle with work-life balance as they shouldered the bulk of childcare. The positive side of lockdown: several women reported a higher quality of life due to less stressful (work and childcare) routines; women's comments on academic work-life balance and childcare outweighed those of men, of whom only two fathers reported being able to spend more time with the children and expressed the joy of fatherhood. No significant gender differences were found in concerns about the end of lockdown.<br><br>*Limitations*: Limited statistical power with only N= 146 responses. *Research team*: respondents' overall quality of life is a more secondary (mental) health outcome; unequal ratio of women to men, limited generalisability of results.
**Balenzano et al., 2020**	The ANOVA shows significant differences between mothers and fathers in the division of housework before COVD-19 ($F(1) = 3.83$; $p \leq .05$; mothers: mean = 1.47; SD= .75 vs. fathers: mean = 1.85; SD= .81) and in the division of childcare, both before ($F(1) = 15.92$; $p \leq .001$; mothers: mean = 1. 43; SD= .82 vs. fathers: mean = 2.25; SD= .79) and during the lockdown ($F(1) = 7.85$; $p \leq .01$; mothers: mean =1.86; SD= .82 vs. fathers: mean =2.05; SD= .89), with mothers having a lower perception of an equal division of unpaid childcare work. In terms of their own and their partner's role during phase 2 of the lockdown (schools and childcare centres remained closed while most parents returned to work), a higher percentage of women reported having the primary responsibility (42% vs. 10%), while a higher percentage of men expected their partners to do so (25% vs. 4%).<br><br>Although respondents were satisfied overall with the division of labour at home and the division of childcare, mothers scored lower on the division of labour at home ($F(1) = 5.62$; $p \leq .05$; mothers: mean = 1.46; SD= .88 vs. fathers: mean = 2; SD= .79) and childcare ($F(1) = 10.17$; $p \leq .001$; mothers: mean = 1.54; SD= .76 vs. fathers: mean = 2.20; SD=.83). Parental stress increased for approximately 50% of respondents during lockdown. More than two-thirds of the parents stated that they had had positive parenting experiences during the lockdown. Although positive parenting experiences and parenting stress were negatively correlated (not significant), no gender differences were found.<br><br>*Limitations*: Limited power of results due to small sample size; possible selection bias as more motivated/positive parents may have participated in the survey; sample consisted mainly of women; use of online self-report and retrospective measures to assess

	outcomes prior to inclusion, limiting the generalisability of results. *Research team*: Limited generalisability of results in terms of gender differences, as only 2 men participated in the study and the overall sample size is very small.
**Bartolj et al., 2022**	There were almost no gender differences in terms of reported paid work time (only 4 minutes less by mothers compared to fathers before the lockdown and no additional difference between them during lockdown); however, there were statistically significant differences in unpaid care work and leisure time, with mothers reporting almost 1 hour more unpaid care work and 1 hour less leisure time before the lockdown, but with no additional differences during the lockdown. Additional gender differences were seen across sectors of employment (private/public) and family-provision status (main/non-main family provider for mothers and fathers): there was almost no difference in paid work hours before the lockdown and a slightly greater adjustment for women during the lockdown. Main-providing women in the public sector reported lower hours of paid work (half an hour more than men; economically significant), with greater gender differences in unpaid care work and leisure time. Along all employment sectors and family-provision statuses, women reported more hours of unpaid care work (between 18 min to 50 min) and enjoyed less leisure time (between 28 min to 58 min less) than men before the lockdown; during the lockdown, these gender differences persisted but did not increase, except for main-providers in the public sector (women increased their time spent on unpaid care work by 1 h 37 min, men by 1 h 9 min). A comparison of the coefficients between employment sectors showed that parents in the public sector did slightly less paid work before the lockdown (between 14 min for non-main-providing fathers and 27 min for main-providing mothers less) and significantly more unpaid work (between 0 min for non-main-providing mothers and 1 h for main-providing fathers more) and less leisure time compared to parents in the private sector; the differences between main-provider vs. non-main-provider was greater in the private sector.<br><br>The descriptive statistics further show that both mothers and fathers seemed to have greater emotional exhaustion during the lockdown than non-parents, while men without children were less likely to be emotionally exhaustion during the lockdown. Econometric analysis revealed a significant increase in the difference in reported emotional exhaustion between parents and non-parents during the lockdown, particularly among parents employed in the public sector and among non-main-providing mothers; differences in hours of paid and unpaid work during and before the lockdown may explain an important part of these differences in changes in emotional exhaustion.<br><br>*Limitations*: Limited generalisability due to potential selection bias and non-random sampling, hence a non-representative sample. Moreover, only a limited set of confounders were used (potential confounding bias); the use of self-reported measures and retrospective pre-lockdown questions may lead to biased results; multitasking as a source of gender inequality was not considered in this study.
**Beno, 2021**	Home-schooling tasks, additional childcare activities, housework and the burden of reconciling this with working from home in general largely fell to women. For all couples, there was a rather stereotypical division of roles, with fathers being the main breadwinners and mothers working part-time, leading to the expectation that mothers were primarily responsible for home-schooling and housework to support full-time working fathers. Gender inequality was mentioned by three out of five women, while two men emphasised gender equality by stating that they tried to share childcare tasks equally to relieve the burden on their wives.<br><br>At the beginning of the lockdown, working from home was perceived as difficult by all respondents; parents of younger children in particular described issues of balancing work and home-schooling as very stressful. Four parents were positive about the lockdown as it meant thy could spend more time with their children. All participants expressed “the fatigue and fear” in relation to the home-office-school environment, which would lead to longer-term exhaustion. Again, all participants acknowledged that a privileged home-office-school environment (i.e., more living space, a job that can be done remotely, or support of the entire family) made it easier to deal with these challenges.

	Limitations: None reported. *Research team*: No information on the interview format; no information on study period, except that the study was conducted in COVID-19 times.
**Cannito & Scavarda, 2020**	Although fathers became more involved in childcare by being more present (at weekends, spending more time at home) and even increased their self-confidence, the division of care work (which was mostly done by women) did not change during the lockdown, especially for less egalitarian couples, which was exacerbated by the lack of external support (e.g. from grandparents or domestic workers).<br><br>Before the pandemic, all parents, especially mothers, described the organisation of childcare and work-life balance as complex and difficult. During the lockdown, family management was perceived as easier and less stressful as there were fewer commutes to work or school, which led to a reduction in parental stress and anxiety. However, working parents reported that the boundaries between (remote) work, personal and family life were blurred; for fathers, family often came into conflict with work, limiting their productivity and leading to feelings of guilt towards employers and colleagues, including high stress and weight loss. For mothers, work interfered with family by preventing them from responding to their children's needs and mentally switching off from their work, leading to feelings of guilt towards their children.<br><br>*Limitations*: Online interviews may lead to a loss of intimacy; small sample size. *Research team*: This is a quasi-pre-post design as the questions were asked at the same time before and after the pandemic, thus not truly longitudinal, which also limits the validity due to recall bias; the sample consisted of couples with high levels of education and good socioeconomic status; limited generalisability to other subgroups of parents.
**Cheng et al., 2021**	Around a third of working mothers spent more than 20 hours per week on childcare and home schooling, compared to less than a quarter of working fathers; 30% of working fathers spent less than one hour per week on these care activities.<br><br>Working people were on average less mentally distressed before COVID-19, with the same results when the sample is restricted to working parents only. Mental well-being worsened by 64% in the first two months of the pandemic among employed parents (mean GHQ-12 Caseness score of 2.72 during the pandemic vs. 1.66 before the pandemic) and by 65% among all employed individuals (2.62 vs. 1.58). The presence of children was not significantly related to mental health, but spending more than 20 hours on childcare or home schooling was associated with worsening mental health; mothers and working parents whose pre-pandemic income was above the median showed the strongest association between child-related activities and mental health.<br><br>*Limitations*: None reported.
**Clemens et al., 2021**	Almost 60% of women said they were the main caregiver for the children during the lockdown (compared to 9% of men who said 'mainly oneself'), and 20% believed this was equally the case (compared to 38% who said 'equally shared'). Men saw their partners as mainly responsible for childcare (45% 'mainly partner').<br><br>The descriptive results showed that women were less satisfied with the division of childcare responsibilities than men, both before (7.8 vs. 8.4) and during the pandemic (7.3 vs. 7.7), with satisfaction decreasing for both genders during the pandemic. ANOVA results showed that parental satisfaction with childcare allocation decreased significantly for all participants during the pandemic compared to before the pandemic, with no significant difference between genders.

	Limitations: The number of male participants is very small; the validity of the findings regarding gender differences in parental satisfaction before and during the pandemic is limited; the questions on parental care before the pandemic and the reports of ACEs are based on retrospective self-reports, as are the reports of ACEs, which limits validity due to recall bias. *Research team*: Only descriptive results were disaggregated by gender, no analyses; parental satisfaction as the main mental health outcome.
**Czymara et al., 2021**	Men were significantly more likely to talk about 'paid work' and 'economy', while women were more likely to mention 'social contacts' and 'childcare', with the most likely comment being written by a woman who expressed an increased need for care and increased work-family conflict during the lockdown. These differences in topic relevance reflected the social situation of both genders, namely one in which the traditional division of paid and unpaid labour follows the male breadwinner model, even among relatively well-educated people. Such gender differences in worry are considered to be part of 'cognitive labour', which is another key dimension of gender inequality in the division of labour within heterosexual couples; the sum of the stress caused by the pandemic and the unequal gender distribution of cognitive labour may be associated with a greater deterioration in job prospects for women than for men and a widening of the gender wage gap during the recovery process. No gender differences were found in relation to the impact of lockdown on 'society' and 'family' in general, nor in relation to 'everyday life' and 'individual concerns'.<br><br>*Limitations*: The positive assessment of their economic situation is due to the fact that respondents were rather young and well educated. *Research team*: High-level recruitment method via official websites and Facebook pages, only in German, which limits generalisability.
**Giurge et al., 2020**	Mega/meta-analyses showed that, overall, there was consistent evidence that women spent more time on necessities (including housework and caring for dependents) during the lockdown compared to men. According to the sample-specific results, women spent more time on care/family time (standardised effect: 0.27**, housework (0.08) and paid work (0.12), while spending less time on total leisure (-0.23**), passive leisure (-0.36**) and active leisure (-0.10). To the extent that women spent more time on housework, they were more likely to report lower subjective well-being.<br><br>Overall, there were conventional significant results regarding time use and happiness for leisure and happiness as a whole (SE = 0.13, P = 0.013); both work and happiness and housework and happiness had negative coefficients. There was neither a significant (negative) effect of gender (1= female) on happiness (ß= -0.05, P = 0.632) nor a significant interaction of parental status on gender differences in happiness (ß= -0.30, P = 0.309), but a significant effect of gender on passive leisure time (-0.35***). Parental status was a moderator in time spent on necessities and work, such that gender differences were larger for parents than for non-parents.<br><br>*Limitations*: It is possible that a significant main effect of gender on well-being was not observed because the negative consequences of additional housework were offset by doing these activities together or the presence of other family members - which was more common during COVID-19 lockdowns. *Research team*: No comparison with pre-pandemic data.
**Hipp & Bünning, 2021**	Women were slightly more likely than men to either have reduced working hours or not work at all during the pandemic, but parenthood was a much more important predictor. While the distribution of childcare was more equal in the initial phase of the lockdown (t1), mothers still performed significantly more unpaid work than fathers over time (t2 and t3). The unequal distribution of unpaid care work varied depending on the parents' current work status and place of work: In constellations where only one partner was in paid work or worked on-site, this partner did less unpaid care work than the one who was not in paid work or the one who worked from home. These patterns were similar for both genders.

	Before the pandemic (t0), there were no significant differences in mothers' and fathers' satisfaction with work, family life or life in general. At the beginning of the lockdown (t1), mothers reported lower levels of satisfaction. Mothers continued to report lower levels of work satisfaction (t3), while their satisfaction with family and life levelled off to pre-pandemic levels.<br><br>*Limitations*: Limited generalisability due to sample selectivity; overrepresentation of women and highly educated individuals and those living in Berlin.
**Ohlbrecht & Jellen, 2021**	Mothers were more likely than fathers to be responsible for childcare during the lockdown (73% of mothers compared to 51% of fathers) and for home schooling (39% compared to 13%). Almost 50% of participants worked from home.<br><br>Parents did not experience a decrease in feelings of stress and exhaustion during the pandemic. Fathers felt happier, more relaxed and more secure than mothers. A total of 72% of parents felt restricted in their everyday lives during the pandemic, including in pursuing hobbies (72%), maintaining social and friendly relationships (92%), maintaining contact with family (86%), volunteer or political activities (52%) and professional activities (47%). Mothers felt more restricted in these areas than fathers.<br><br>*Limitations*: Limited generalisability due to the selectivity of the sample; overrepresentation of younger and more highly educated people. *Research team*: Respondents were interviewed at a single point in time; pre-pandemic data refers to retrospective responses.
**Xue & McMunn, 2021**	In both waves (April and May 2020), women spent more time on housework (approx. 15h vs. <10h) and childcare/ home-schooling (20.5h/22.5h vs. approx. 12h) than men. In couples, women accounted for 64% of housework and 63% of childcare/ home-schooling. Mothers were more likely to reduce their working hours (21% mothers only vs. 11% fathers only) or adjust their working hours (32% mothers only vs. 18% fathers only) due to childcare/ homes-schooling; in contrast, only 4% of couples reduced their working hours and 14% adjusted their working hours.<br><br>In April, increased housework and childcare/ home-schooling were only associated with a higher level of psychological stress in women (relatively weak association); no significant association was found in men. Apart from the discontinued association between hours of housework and GHQ-12 Likert scores among women, similar associations were observed in the May wave. For couples, women's share of unpaid care work was not associated with either men's or women's GHQ-12 Likert scores in either wave. Single mothers who adjusted their work hours due to childcare/ home-schooling had on average 3.93 higher GHQ-12 Likert scores than single mothers who did not; adjusting work hours was not related to the GHQ-12 Likert scores of coupled mothers.<br><br>*Limitations*: Analyses did not assess the change in unpaid care work between pre-lockdown and lockdown and how this change affected psychological distress; COVID-19 online surveys had a lower response rate (approx. 40%) than the main annual survey; possible bias due to missing data (complete case analysis).
**Yerkes et al., 2020**	Mothers were more likely to work in essential occupations than fathers, reported more adjustments to working times, and experienced both more and less work pressure in comparison to before the lockdown. They continued to do more childcare and housework than fathers, although some fathers reported taking on a greater share of childcare and housework during the lockdown than before.<br><br>In terms of childcare, 17% of parents reported an increase in care tasks, with the share of fathers (22%) being significantly higher than mothers (12%). Around 20% of parents reported doing less care tasks relative to their partners during the lockdown compared to before, with only slight differences between mothers and fathers. More fathers reported an increase in the relative share of care

	tasks during the pandemic compared to before, however the overall division of childcare between mothers and fathers remained unequal: 60% of mothers reported doing (much) more childcare tasks than their partners during lockdown (vs. 10% of fathers). Thirty-four percent of parents reported an equal division before lockdown; during the lockdown, this remained the same for 35% of parents. Parents working in essential occupation had significantly smaller chances of doing more childcare tasks during the lockdown than before (13%). With regards to housework, 12% of parents reported doing more housework during the lockdown than before, with only 10% of fathers reporting doing much more than their partners. Around 32% of parents reported an equal share of household tasks during lockdown; the increase in fathers' relative share was significant. While, overall, mothers continued to do relatively more than fathers, fathers had a significantly greater chance (10%) than mothers to report an increase in the relative share in household tasks during the lockdown. Occupation had a larger effect than gender, but only for parents who reported doing less housework during the lockdown than before.<br><br>In terms of quality of life, mothers reported a greater decline in leisure time than fathers (57% vs. 36%); the gender difference was significant. Parents with essential occupation were more likely to struggle with reduced leisure time than parents without essential leisure occupation. Having children in primary school was also a significant explanatory factor for differences in leisure time. Deterioration in work-life balance was equally pronounced among all parents; gender was an insignificant covariate/ control variable, while the educational level and schooling stage of children appeared to explain the variation in perceived work-life balance during the lockdown. No changes in relationship dynamics were observed for most parents (no gender differences). A significant increase in disagreements between parents about the division of childcare tasks was observed (no gender differences). Parents with children in primary school were more likely to report an increase in disagreements about the division of childcare tasks (13%) than parents with children in secondary school.<br><br>*Limitations*: The lack of data on the age of the youngest children does not allow for a distinction between children who are not yet of school age and children who have already completed secondary school. There is a lack of data on the outsourcing of household or childcare tasks. *Research team*: There was an opportunity to describe changes not only by gender and occupation, but also at the intersection of both categories, but the authors did not do so. No comparison group (i.e., non-parents).
**Zhou & Kan, 2021**	Housework hours increased during lockdown and peaked in April and May 2020 (12.3 hours/week), then gradually decreased and remained at 10.5 hours/week thereafter. Compared to the lockdown in September 2020, the lockdown in January 2021 was not associated with an increase in housework time; rather, a relatively high level of paid work time was observed in the latter two lockdown periods. No gender differences were observed for housework time.<br><br>The increase in distress levels was much greater for women in the first month of the first lockdown ($p < 0.001$); thereafter, women's subjective wellbeing recovered, whereas men's distress levels began to rise. Distress levels for both gender groups were lowest from July to September 2020 (return to "normal life"). With the re-imposing of lockdown measures from November 2020 to January 2021, distress levels rose again more sharply for women than for men. Overall, women's distress levels reached a similar peak across the three lockdowns, while men's distress levels were higher during the later lockdowns than during the first lockdown (with an increase in COVID-19 cases and related deaths).<br><br>*Limitations*: The COVID-19 sample is socioeconomically advantaged in terms of employment, occupation, education, and homeownership compared to the overall UKHLS sample, so the results may underestimate the potential negative impact of COVID-19 and the related lockdown. *Research team*: No descriptive statistics (weighted data only).
Grey literature	

Bolis et al. 2020	In the UK, nearly half of women said that unpaid care and domestic work had increased (39% of women said their unpaid care work had increased by more than 3 hours), and 36% of men said their time spent on unpaid care work (care and domestic work) had increased. Compared to other countries, British men spent relatively less time with meal preparation and cooking, which takes up the most time for 19% of the men surveyed. 17% of British women said that childcare was one of their most time-consuming daily tasks (which is relatively low compared to other countries), which may be due to parents in high-income countries relying on technology for schooling and child entertainment. The men surveyed stated that they wanted to spend more time with their children due to the increased time spent at home during the lockdown. When asked if they thought men should do more unpaid care and domestic work in the household, 76% of women and around 60% of men said yes.<br><br>Women were more likely than men to report feeling anxious or depressed. Key workers were more likely to feel stressed and anxious than non-key workers.<br><br>*Limitations*: Survey was relatively short; interesting intersectional dimensions could not be considered due to lack of data collection (e.g., information on ethnic background). The increase in unpaid care work was captured by estimated ranges (e.g., up to two hours, two–three hours, etc.). *Research team:* No further specification of the mental health item; no indication of absolute or relative numbers.
Bujard et al., 2020	On average, men spent 1.9 hours on daily family work compared to 3.2 hours for women in 2018. In April 2020, women spent 3.4 and men 2.5 hours on family work on average. Time spent on family work did not substantially differ pre to during pandemic; there was a substantial increase for parents, most probably due to additional care work due to home-schooling and childcare. For mothers, daily average family time increased from 6.6 in 2018 to 7.9 hours in April 2020 compared to 3.3 to 5.6 hours for fathers. Parents of under 6-year-olds spent more time on family work than parents of over 6-year-olds, however the average time spent on family work has increased for both parent groups from 2018 to 2020, with the highest increase being for fathers of under 6-year-olds with an increase of 2.6 hours. Short-time working or furloughed fathers took on a large share of family work during the crisis, whereas in other constellations mothers took on a much larger share of family work than fathers. Regardless of the work arrangement (both working from home, one working from home and other onsite, one working onsite partner working from home, both onsite), mothers spent more time on family work than fathers, e.g., working-from-home mothers with partners working onsite spent 10.2 hours on family work compared to 5.0 hours for men in the same situation. If both parents worked onsite, mothers spent 8.6 and fathers 5.1 hours on family work on average in April 2020. This is also in line with women being more likely to reduce work hours than men.<br><br>There was little to no change in work satisfaction from 2019 to 2020, both for men (6.4 points) and women (6.4 to 6.3 points). There was no decline in life satisfaction for fathers, but a minor decline for mothers (6.6 to 6.2). Short-time working men had the lowest work satisfaction levels (5.6 for men without children in household, 5.4 for fathers) and were the only ones with a decline in satisfaction levels over time among all men; working-from-home and working-onsite men were more satisfied (6.7). There was a greater decline in work satisfaction for women; regardless of work arrangement and children in household, satisfaction levels declined for all women from 2019 to 2020. Short-time working women without children in household showed the lowest satisfaction levels (5.8 in 2018 and 5.0 in 2020). Mothers of over 6-year-olds had the lowest work satisfaction with 6.1 points during the pandemic, whereas fathers had an average work satisfaction of 6.7; this hints towards a higher involvement of mothers in home-schooling and mothers being interrupted while working during the day.<br><br>Regardless of gender and children in household, there was a considerable decline in family life satisfaction levels from 2019 to 2020, from 7.4 to 7.0 for men and from 7.7 to 7.0 for women. Considering work arrangements and children in household, short-time working fathers were the most satisfied (7.6), whereas mothers in the same situation had a family life satisfaction of 6.9 points. Working-from-home mothers were more satisfied than working-from-home fathers (7.4 vs. 7.1) and working-onsite mothers (7,4 vs. 6.9). There was no difference in satisfaction levels for working-from-home fathers (7.1) and working-onsite fathers (7.2). Among

	persons without children in household, working-from-home and short-time working persons were comparatively dissatisfied with family life (except for short-time working women). Family life satisfaction decreased from 2019 to 2020 for working-from-home men (by 0.7 points for those with children, and by 0.4 for those without children). Working-from-home fathers were most satisfied with family life among all groups. Women's satisfaction levels decreased for all groups, especially for working-from-home women without children and working-onsite mothers (each by 0.8 points). Working-from-home mothers in April 2020 were the most satisfied among all groups. Working-from-home women and working-onsite or short-time working mothers were the least satisfied among all groups and time-points. Mothers with over 6-year-olds had the highest decline (from 7.7 to 6.7 points), whereas fathers were more satisfied (7.0) and had a lower decline (by 0.4 points). Among parents of under 6-year-olds, satisfaction levels declined slightly more for fathers than for mothers.<br><br>*Limitations*: None reported.
Chung et al., 2020	Before the pandemic, the proportion of couples with children under 18 where women did most/all unpaid care work was between 52-65%. During the pandemic, the proportion fell to 44-56% for all indicators (the biggest change was in cleaning/laundry from 65% to 56%), indicating a more even distribution of unpaid care work during the lockdown. An exception is 'DIY, maintenance, transport' with shares of 12% before the pandemic and 14% during the pandemic, which is most likely due to this area being seen as more male-dominated. Cleaning (65%), routine childcare (60%) and education (60%) had the highest shares. There was a large discrepancy between mothers and fathers when asked what they considered to be 'equally sharing care': 65% of all fathers said that they shared or were responsible for unpaid care work, compared to 47% of mothers who said this was the case. Most women said they did (much) more cooking (65% vs 45%), cleaning/laundry (54% vs 44%), routine childcare (69% vs 63%), non-routine childcare (68% vs 61%) and education (76% vs 58% for men), with a greater proportion of mothers saying they did 'much more' than 'more' childcare work.<br><br>Parents were generally more likely than non-parents to report feeling rushed/pressed and nervous/stressed, with the proportion highest among mothers (48/46% compared to 35/32% for fathers). The difference between the genders was also evident among non-parents (37/46% of women compared to 35/32% of men). In terms of the positive aspects of lockdown, most respondents said they felt 'happy and in good spirit' (approx. 52-61%), followed by 'calm and relaxed' (more common among men and fathers), their lives were interesting (much more common among non-parents, especially men, least common among mothers), 'active and vigorous' (less likely among women, least likely among mothers) and 'fresh and rested' (much more common among non-parents, especially men; less likely among women, especially mothers). Mothers were more likely than fathers to say that their job prevented time for family (48% vs. 40%) and that family prevented time for job (49% vs. 32%). Compared to the 2015 EWCS data, fathers perceived more work-family conflict in 2015, while mothers perceived more work-family conflict during the lockdown (women also tend to reduce their working hours during the lockdown).<br><br>*Limitations*: As data was not available for all couples in the datasets, a direct comparison was not possible. *Research team*: Unclear sample size; the methodology of data collection is not specified in the report and had to be determined by contacting the corresponding author.
Close the Gap & Engender, 2021	Women's employment was disproportionately affected by the additional caring responsibilities, while women were particularly affected by increased financial insecurity and anxiety during the COVID-19 pandemic. Seventy-two per cent of mothers said that housework was mainly done by them, while 4% said it was mainly done by their partner and 24% said it was done equally by both. Sixty-one per cent of women said they did most of the shopping themselves, while 10% said it was mainly done by their partner and 28% said it was done equally by both.

	Mothers were more likely than fathers to report high anxiety levels (36% compared to 29%). Compared to 28% of white mothers and fathers, 34% of BME mothers and 32% of BME fathers reported high levels of anxiety. Compared to non-disabled mothers (32%) and non-disabled fathers (24%), 58% of disabled mothers reported high levels of anxiety.<br><br>*Limitations*: None reported.
Destatis, WZB & BiB, 2021	At the beginning of the pandemic in April 2020, women were slightly more likely to work from home than men. Working mothers spent less time on paid work, both on site (6.6 vs. 8.3 hours per day) and at home (5.2 vs. 7.0 hours per day) compared to working fathers, suggesting that working mothers were more likely to work part-time. Working mothers spent more hours per day on home and family work (8.2 on site, 8.0 from home) than working fathers. Fathers working from home appeared to have spent the time saved (no commuting, taking children to school/daycare etc.) on home and family work (5.5 vs. 4.9 hours daily). Overall, women spent more time on family work than men, regardless of their work arrangements.<br><br>In terms of satisfaction with work (6.7 vs. 6.7) and family life (7.2 vs. 7.1), no differences were found between fathers who worked from home and fathers who worked on site. Mothers who worked from home were slightly more satisfied with their work (6.6 vs. 6.2) and family life (7.4 vs. 6.9) than mothers who worked on site, who had the lowest job satisfaction (6.2) of all groups. This suggests that these working mothers had a greater conflict between work and family during the lockdown. At the same time, the mothers appeared to have been much more reliant on working from home, as they were the main carers in most households. To summarise, mothers who worked from home were more satisfied with their work and family life, suggesting a positive impact of home office on work-family balance for women (working from home is not always possible and depends on employment).<br><br>*Limitations*: None reported.
Etheridge & Spantig, 2020	Around 54% of respondents had poorer mental well-being in 2020 than in 2017, 61% of whom were women. The gender composition was the same for respondents whose well-being had not changed (11%) and those who had better well-being in 2020 (34%). Wellbeing by gender and time spent on childcare (all respondents, with and without children) indicated that all those with childcare responsibilities prior to the pandemic had poorer wellbeing in 2020, with no significant changes among those with moderate childcare responsibilities (1 to 15 hours/week) and those with no childcare responsibilities. Only those with significant childcare responsibilities (15+ hours/week) were significantly worse off; across all genders, women were particularly affected (70% of those with significant childcare responsibilities were women; 14% of all women, 8% of all men). A similar picture emerged for time spent on housework: the average mental wellbeing of all groups deteriorated in 2020 compared to before the pandemic, although the decline in wellbeing appeared to decrease further with increasing housework for women, but not for men. There is an imbalance in the gender distribution across these categories: 39% of men reported doing less than 6 hours of housework, while only 20% of women reported the same. More than 50% of all women surveyed reported doing a significant amount of housework (10+ hours/week), compared to 29% of men. In terms of caring for others outside the household, both groups showed a decline in wellbeing in 2020; again, there is a gender imbalance in caring responsibilities (51% for women, 45% for men). Linear regression models: Women experienced a 0.14 SD greater decline in well-being than men during the pandemic, controls for housework and caring tasks reduced the original estimate of the gender difference by 0.03 SD or 22% (increasing the gender difference), whereas financial controls appeared to reduce the gender difference as well as loneliness and number of close friends (reducing the difference by 67%, leaving only 0.05 SD of the original difference unexplained), suggesting that women reported a greater number of close friends before the pandemic and greater loneliness after the pandemic. In summary, much of the gender difference in well-

	being could be explained by gender differences in social factors and increased feelings of loneliness, although gender differences in family-related time allocation and caring responsibilities also played a role.<br><br>*Limitations*: None reported.
Eurocarers/IRCCS-INRCA 2021	Women and Italian carers provided care for longer than other groups; the men surveyed had better health than the women (58% 'good or very good' compared to 48% of women); 61% of carers stated that their health was negatively affected by caring (higher in Germany at 76%, lower in Portugal at 55%), significantly higher among the women surveyed (64% compared to 48% of men). 48% of women surveyed (64% compared to 48% of men); the main recipients of care were spouses or partners (30%), and the most common chronic illness or health condition underlying caregiving tasks were physical disabilities (70%), followed by cognitive impairments (e.g. Alzheimer's and dementia at 51%).<br><br>The average number of informal care hours per week increased from 46.6 before the pandemic to 54.5 (+17%), with women experiencing a greater increase than men (from 47.9 to 56.5 compared to 39.2 to 45.2 average weekly hours); during the pandemic, informal carers (especially women) had to intensify their care activities, in particular emotional support (60. 3 % of all cases), remote communication (49.7 %), practical help on site (43.9 %), care coordination and support (43.7 %) and help with transport (37.7 %) - women reported a higher intensification in all these activities.<br><br>Sixty-nine per cent of women reported a worsening of their mental health/ psychological state of mind (compared to 57% of men), with a gender difference of +12% (better for men), followed by 'overall health status' (+11.4%) and 'physical conditions' (+9.3%).<br><br>*Limitations*: None reported. *Research team*: Percentages are based on the total study population; no further specification of mental health measure.
Eurofound 2020	Women were generally more involved in caregiving (35 hours vs. 25 hours per week for men) and housework activities (18 hours vs. 12 hours per week for men) in July 2020, with gender differences in weekly hours across Europe (caregiving: 1-2 hours difference in Belgium, Germany, Slovenia and Spain vs. 26 hours difference in the Netherlands; housework: 2-3 hours difference in Denmark, Finland, France and Sweden vs. 13-14 hours difference in Romania and Greece). The gender-specific differences in caregiving and housework hours increased even further among respondents with children under 12: women spent 62 hours per week caring for children (compared to 36 hours for men) and 23 hours on housework (compared to 15 hours for men). Single parents spent an above-average number of hours on childcare (52 hours for women, 36 hours for men); single women with children under the age of 12 spent the most hours (77 hours per week). Employed women with children under 12 in the household spent 54 hours a week on childcare (compared with 32 hours for men), while unemployed or inactive women spent 74 hours (compared with 37 hours for men). Both employed (16 vs. 11 hours per week) and unemployed/inactive women (20 vs. 12 hours per week) spent more time on housework than men.<br><br>In April 2020, young respondents and the unemployed reported the lowest levels of mental well-being. Overall, the mental wellbeing of EU respondents improved between April and July from a WHO 5 score of 49 to 53. The largest increase was among those aged 50+ (50 in April vs 55 in July) - younger respondents still reported lower mental wellbeing. Women's mental wellbeing was lower than men's in both April (47 vs 51) and July (51 vs 54), although both genders showed an overall improvement. Gender differences in loneliness were small (13% of women versus 12% of men in July compared to 16% for both in April), with greater differences between age groups (the youngest were more likely to report loneliness).

	Limitations: Snowball-sampling resulted in a non-representative sample, but it was weighted; potential self-selection bias due to recruitment via social media and snowballing, with different response rates across European countries. *Research team*: The study sample consists of participants from all over the world (approx. 5 % from non-EU-27 countries); sometimes it is unclear whether the reported results refer to the total sample or to the EU-27 sample.
Hübgen et al., 2021	On average, women spent more time on childcare and housework than men. Women were more likely than men to report being entirely/mainly responsible for childcare; before the pandemic, their propensity was 10% higher than that of men, with the gender gap widening over the course of the pandemic (13% in March 2020, 15% in May 2020). Women predominantly took on childcare when the youngest child was between 0 and 12 years old, with a 19% higher propensity than for children between 13 and 18 years old. Parents with a university degree were more likely to share childcare more evenly; before the pandemic, the propensity was 11% higher, but the difference narrowed during the pandemic: academic mothers were more likely to take on additional childcare responsibilities in full than academic fathers; however, childcare was more evenly shared among academic parents than among parents with a lower level of education. Employment status and WFH: Non-employed and WFH partners were more likely to provide the majority of childcare. If both parents were WFH: Before the pandemic, the propensity to share childcare equally was 19% higher; the propensity decreased over the course of the pandemic, eventually reaching pre-pandemic levels in March 2021; the propensity increased for mothers with WFH in March, May and June 2020, and remained the same for fathers. This pattern was similar for the division of housework: mothers showed a higher propensity to do most/full housework at all time points (21% higher before the pandemic, 3% in March 2020 and 25% in May 2020). At the beginning of the pandemic, academics showed a 10% higher propensity to share housework equally compared to respondents with a medium or lower level of education, with the difference decreasing as the pandemic progressed; among academic mothers, the propensity to take on all/most of the housework increased. When both parents were working, fathers were 9% more likely to do all/most of the housework than parents who worked from home; over the course of the pandemic, the tendency to share housework equally increased in this constellation. When only women did WFH, they were 9% more likely to do housework than parents who worked on site. When only men did WFH, they had a 5% higher propensity, with partners in this constellation tending to share housework more equally. There is a clear gender difference in mental health across all waves, with women more likely to report anxiety/nervousness/restlessness and hopelessness/stress (up to 15%), with the difference being greatest in March 2020 compared to men. This gender gap narrowed as the pandemic progressed, but women still tended to have a 6% higher propensity to experience mental stress in March 2021 (the first lockdown appeared to be the most difficult for women in terms of multiple burdens, mental and physical health). Overall, women were less satisfied with their work than men before the pandemic, but more satisfied with family life and life in general. Women's satisfaction in all three areas worsened over the course of the pandemic compared to men; in March 2020, women had the lowest satisfaction scores.<br><br>*Limitations*: The propensity of Berlin couples to share childcare more equally was 10% higher than in other regions of Germany; this decreased over the course of the pandemic. Berlin fathers were more likely to report being wholly or mainly responsible for childcare; Berlin mothers showed a higher propensity to be the sole carer than Berlin fathers at the beginning of the pandemic, with a lower propensity in May and June 2020. The same was true for housework, but with no significant differences in terms of mental and physical health.
Illing et al., 2022	The time spent on childcare increased for all parents during the first lockdown, especially for mothers and fathers with children under 13. Mothers with young children showed the highest increase in absolute numbers: Mothers with children aged 6-12 saw an increase of 10 hours between February and April 2020, while fathers saw an increase of 7 hours. As fathers initially provided less childcare, they recorded a higher relative increase in the same period (83% compared to 61% for women). In April 2020, mothers with children aged 6 years or younger spent around 8 hours more on childcare than in February, compared to 6 hours for fathers. After the first lockdown in September 2020, the amount of time spent on childcare decreased for both genders; parents of children aged 12 or younger spent an additional 2 hours per week on childcare compared to February 2020. Overall, working mothers spent more time on childcare, while the paid working hours of fathers decreased more than those of mothers. Working mothers with

	children aged 12 and under had a higher total workload (the sum of paid work, childcare, housework and commuting time) than working fathers, with an increase of 8 hours per week in spring 2020 compared to 3 hours for men. The total workload of mothers with young children increased by 6 hours per week in April 2020; the total workload of fathers also increased significantly. Parents with school-age children recorded the highest increase in absolute weekly hours; in September 2020, the total workload almost reached the initial level of February 2020. The higher overall burden on mothers had a negative impact on their life satisfaction. Satisfaction levels fell for all respondents during the first lockdown, but the decline was greatest for mothers with children aged 12 or under. For fathers, there was no difference in the development of life satisfaction between the three groups (no children/older children, children aged 6 or under, children aged 6-12). In September 2020, when the overall burden was balanced again, there remained no different trends in life satisfaction.<br><br>*Limitations*: None reported. *Research team*: No further specification of the mental health measure.
Kalaylıoğlu et al., 2020	Although there were no notable gender differences, more women reported spending more hours on household activities, with the proportion of women reporting increased workloads highest in the categories 'cleaning (the household and washing clothes)' (77.6% compared to 47% of men) and 'cooking and serving meals' (59.9% compared to 23.9% of men). In all other categories (e.g. 'caring for children, including feeding, cleaning, physical care'), the proportion of women (23.8%) and men (20.2%) who reported an increase in workload was fairly similar. Among those who stated that they 'do not usually do' certain household activities, the gender-specific distribution of unpaid care work was clearer in the categories 'cooking and serving meals' (5.0% of women compared to 40.7% of men) and 'cleaning and maintaining own dwelling and surroundings (e.g. clothes, household)' (2.2% compared to 25.5%). In terms of the household activities that participants spent the most time on since the spread of COVID-19, it is noticeable that women spent the most time on 'cleaning and maintaining own dwelling and surroundings (e.g. clothes, household)' (56.1% compared to 27.0% of men) and 'cooking and serving meals' (24.6% compared to 6.3% of men). 'Shopping for my family/household member' was the household activity that men spent the second most time on (24.6% compared to 5.0% of women), followed by 'Decorating, repair and household management (e.g. paying bills)' (17.6% compared to 17.6% of women), "playing with, talking to and reading to children" (10.9% vs. 3.0% of women) and "affective/emotional support for adult family members" (6.3% vs. 1.8% of women).<br><br>Although men were taking on more responsibility in the household, women continued to do most of the housework. In terms of changes in roles and responsibilities within the family since the spread of COVID-19, the proportion of women who reported changes was highest for 'my partner helps me more with household chores and caring for family' (35.2% 'yes', 13. 0 % 'no'), followed by more help from other family/household members apart from children and partner (29.6 % 'yes', 14.9 % 'no'), more help from daughters (23.6 % 'yes', 8.8 % 'no') and more help from sons (15.2 % 'yes', 14.9 % 'no'). Among the men who indicated changes in household tasks and responsibilities, the proportion of those who indicated more help from their partner was highest (36.4 % 'yes', 8.4 % 'no'), followed by family/household members other than children and partner (26.8% 'yes', 17.4% 'no'), more help from daughters (20.3% 'yes', 10.0% 'no') and more help from sons (17.5% 'yes', 11.6% 'no'). In general, the partner seemed to be the biggest support for both women and men during the COVID-19 period, followed by other family members, daughters and then sons. The higher proportions of daughters who help more compared to sons who help more indicate a gender-specific division of roles and responsibilities in the household among children who (have to) take on caring tasks during COVID-19.The proportion of women (54.0%) and men (49.0%) who reported that their psychological/mental/emotional health (e.g. stress, anxiety, etc.) was affected by COVID-19 differed only slightly, with slightly more women reporting a deterioration in their mental well-being.<br><br>*Limitations:* None reported.
OECD 2021	Mothers of under-12-year-olds were almost three times as likely as fathers to say they take on all or most of the additional childcare responsibilities (61.5% of mothers compared to 22.4% of fathers), although these gender differences were the same across the ages of the under-age children. This difference between the genders was smallest in the Netherlands, where it was still 15.9 percentage

	points. Of fathers, 25.9% said that their partner took on the majority/most of the additional care work; the most common response among fathers of under-12-year-olds was that the work was shared equally between them and their partner (40.8% compared to 20.7% of mothers). Gender inequality in unpaid care work was negatively associated with women's employment: a linear regression analysis shows that a high unpaid care workload is highly correlated with leaving paid work (of course, not being employed means free time that can be used for unpaid care work, a high unpaid care workload can push people out of paid work). Working mothers with a working partner were more than twice as likely as working men with a working partner to state that they have taken on most/all of the additional childcare responsibilities (53.4% compared to 20.9% of fathers). The gender-specific care gap was greatest when the mother was not employed and the father was employed: 76.9% of mothers in this constellation stated that they took on the majority/most of the additional childcare tasks, compared to 24.5% of fathers in the same situation.<br><br>Women were more likely than men to report a deterioration in mental well-being (for themselves or a household member). Systemic level: In countries facing prolonged school closures, there tended to be greater gender differences in the distribution of additional unpaid care work (e.g. Türkiye had the longest days of school closure and the highest gender care gap, followed by Poland and Ireland; Norway had the shortest days of closure and was among the countries with the lowest gender care gap; Portugal as an exception with rather short school closure days and the highest overall gender care gap, the Netherlands with medium length closures and the lowest gender care gap). At the same time, the gender care gap was lower in countries that spent more on family support (e.g. Norway and Denmark compared to Türkiye and Portugal).<br><br>*Limitations*: With regard to mental well-being, it is not possible to determine the extent to which respondents were referring to themselves or a household member.
Tani et al., 2021	Working mothers tended to spend more than 20 hours on childcare/homework (34% compared to 23% of working fathers), followed by 0 hours (28% compared to 31% of working fathers), 7-20 hours (21% compared to 20% of working fathers) and 1-7 hours (17% compared to 26%). Before the pandemic, both working individuals and working parents were less anxious. With the outbreak of the COVID-19 pandemic, the mental well-being of working parents deteriorated by 64%; the mental health of working parents was worse than that of working individuals in the sample. Financial insecurity predicted a deterioration in mental health both for households below and above the median income in the pre-pandemic wave and for mothers and fathers in the sample; the relationship was stronger for poorer households and for fathers. The presence of children per se was not significantly associated with mental health; however, spending more than 20 hours per week on childcare/home schooling was associated with a deterioration in mental health: Working parents who spend more than 20 hours on childcare/home-schooling had a 0.525 higher Caseness score compared to working parents who spend less than one hour on childcare/home-schooling; working parents whose household income was below the median (pre-pandemic) showed no significant association between time spent on childcare/home-schooling and mental health. Mothers and working parents whose household income was above the median (before the pandemic) showed the strongest relationship between childcare/home schooling and mental health: mothers tended to find childcare more stressful than fathers (0.580 vs. 0.404 coefficient estimate for '20+ hours' for fathers). Overall, working mothers (compared to working fathers) and working parents from poorer households (compared to working parents from higher-income households) had poorer mental health and financial situations.<br><br>*Limitations*: None reported.
The Fawcett Society et al., 2020	Women reported doing the most housework (73% BAME women compared to 53% BAME men, 74% white women, 52% white men) and the most childcare (74% BAME women compared to 51% BAME men, 73% white women compared to 46% white men) during the pandemic, regardless of ethnicity/racialisation; the least unpaid caring work was reportedly done by white men. Gender differences in responses were relatively equal for BAME and white people. BAME women were most likely to say that they struggled

	to balance paid work and childcare (57%), managed all the different demands on their time (57%) and went shopping or did other tasks at home because of their children (50%), followed by white women (46/50/48%), BAME men (46/50/47%) and white men (34/37/33%). The gender gap in responses was greater for white people than BAME people. In terms of informal caring, BAME people overall (particularly BAME women at 24%, followed by BAME men at 22%) reported spending more time caring for other adults (compared to 10% and 15% less time respectively). White people were less likely to report spending more time on informal caring: 18% of white women and 17% of white men (compared to 7% less time each). Forty-two per cent of respondents found it difficult to cope with social isolation, with white men least likely to do so; the level of coping was not statistically different between BAME men (42%), BAME women (41%) and white women (45%). Compared to the secondary data from 2019, life satisfaction and happiness were significantly lower for all respondents, while anxiety was significantly higher. Life satisfaction and happiness scores were lowest for BAME women (5.1 and 5.3 respectively compared to 5.4 and 5.4 for white women) and highest for white men (5.5 and 5.6 compared to 5.3 and 5.4 for BAME men). Anxiety scores were highest among BAME women (5.4), followed by white women (5.3); BAME and white men had the lowest anxiety scores at 4.6 each. White men were most likely to have high levels of life satisfaction and happiness (scores of 7 to 10; 42% and 43% respectively), and along with BAME men (42%) were least likely to have high levels of anxiety (43%). BAME women and white women were also the least likely to have high life satisfaction (33% vs. 35%) and high happiness (36% vs. 34%), and similarly likely to have high anxiety scores (50% each).<br><br>*Limitations*: Only statistically significant results were reported.
Zoch, Bächmann & Vicari, 2020	Around 57-86% of respondents stated that mothers were the main caregivers to compensate for the closure of schools and childcare facilities. In families with a school-age child aged around 14, only 18% of fathers contributed to family work; for parents with younger children (under 14), the proportion of fathers involved was higher (35-70%). Most families did not rely on other relatives and did not have access to emergency care during the first months of the pandemic, with the exception of highly educated families (relatives and others were more likely to be involved in childcare (24-28%) and children were more likely to attend formal emergency care (8-19%)). Both overall satisfaction and satisfaction with family life were higher in all subsamples before the pandemic, with satisfaction decreasing for both genders, but more so for mothers than fathers. Multivariate results: The results of different models suggest a traditional division of childcare in German families during the peak of the COVID-19 crisis, with families often relying on exclusive care by the mother. The results also point to systematic differences in families' childcare arrangements depending on prevailing gender roles as well as parents' current working conditions (e.g. working from home, key worker status, changing working hours). More traditional gender roles were associated with less paternal involvement in childcare, while the likelihood of exclusive maternal care increased. Fathers' remote working had a significant impact on whether mothers acted as exclusive carers; for mothers, key worker status, longer working hours or working locally rather than remotely were not associated with a higher likelihood of paternal or shared care. Appropriate working conditions appeared to have a positive impact on bargaining power, particularly for fathers but not for mothers. With regard to parental well-being, there were no significant differences in parental satisfaction depending on the chosen childcare arrangement.<br><br>*Limitations*: Small sample size, especially for mothers and fathers with young children; further subsample analyses were not possible. Risk of biased estimates remains due to unobserved characteristics (e.g., partner characteristics, which are likely to correlate with respondents working conditions and chosen care-arrangements).

Notes: ACEs = Adverse Childhood Experiences. BAME = Black, Asian and Minority Ethnicity. BME = Black and Minority Ethnicity. EWCS = European Working Conditions Survey. GB = Great Britain. GHQ = General Health Questionnaire. UKHLS = Understanding Society: UK Household Longitudinal Study. SD = Standard Deviation. WFH = Working from home.
