# Supplementary material for: Caring is not always sharing: A scoping review exploring how COVID-19 containment measures have impacted unpaid care work and mental health among women and men in Europe
Source: PLoS One. 2024 Aug 30;19(8):e0308381. doi: 10.1371/journal.pone.0308381 (PMC11364293; doi:10.1371/journal.pone.0308381)
Supplement: S4 Table — (PDF) [file pone.0308381.s004.pdf]

Caring is not always sharing: A scoping review exploring how COVID-19 containment measures have impacted unpaid care work and mental health among women and men in Europe

S5 Table. Inclusion of gender and intersectionality

Reference	Gender in title or objectives?	Conceptualisation of gender & gender (in-)equality	Mentioning of intersectionality[1]
Peer-reviewed research articles			
Ashencaen Crabtree et al., 2021	Neither	Gender binary;<br>Elaborate discussion on gender in the context of neo-liberal ideologies and practices in Higher Education (HE): assumption of worker "carelessness" (being free from domestic care commitments, which explains the lack of women leaders in HE); indicators of stronger gender bias irrespective of the normativity of socially constructed gendered, personal responsibilities; research evidence regarding women's likelihood of undertaking less valorised academic tasks; reference to how women in science are shoved out of the public debate on COVID-19 explained by "cultural sexism" while research funds for COVID-19 studies go more to men with acute gendered discrepancies in journal article production; lockdown seems to have exaggerated gender imbalance in the "masculinist work environments" of HE institutions related to the "ideal worker" notion; the social construction of time or rather "timelessness" assumes that time will not be punctuated by feminized domestic commitments and associated interruptions of time, much in line with ideal worker norms that expect women to approach work as though they do not have unpaid care work commitments vs. intensive mothering norms that expect women to parent as if they do not have careers.	Not reported
Balenzano et al., 2020	Neither	Gender binary;<br>Discussion of gender inequality at the intersection to cultural narratives and realities in Italy; review of pre-existing gender inequalities in terms of work-life balance difficulties, gender employment gap and gender care gap, reduced female employment rates (especially in Southern Italy) compared with other European countries; discussion of "crisis of family" and family support in the context of Italian cultures and norms of caretaking and social	Implicit;<br>Analysis of gender differences with respect to family SES; reported as not significant.

		support; discussion of a possible lockdown-induced reproduction of Italian culture of male-breadwinner model.	
Bartolj et al., 2022	Neither	Gender binary;<br>Reference to gender gaps in unpaid care work in background section embedded into the cultural context of the setting Slovenia.	Not reported;<br>Subgroup analyses [gender x employment sector (private/public) x family-provision status (main provider/non-main-provider)]
Beno, 2021	Part of research question	Gender binary;<br>Reference to "role theory" to explain the persistence of the male breadwinner model among respondents.	Not reported
Cannito & Scavarda, 2020	Both	Gender binary;<br>Elaborate consideration of gendered dynamics, especially within families and workplaces, gender norms and gender roles/ gender attitudes that are incorporated in society, in the nuclear family and in workplaces as "gendered organizations" that promote and reproduce gender inequalities in terms of paid work, the distribution of care work, and work-life balance; reference to different gender-related theoretical models ("involved fatherhood model", "boundless job model", "new fatherhood", "good fatherhood"); discussion of workplaces and companies as "gendered organizations" that incorporate and reproduce harmful gender attitudes by marginalizing fathers' work-life balance needs in company-level policies; elaborate discussion of traditional gender roles/ "femininity" and "masculinity", both in general and embedded in the cultural setting of Italian individuals (parents, workers, working parents); discussion of work-life balance, "boundless work", "role overload", flexible work and their impact on working mothers' labour market participation and their wellbeing; discussion of working from home and its impact on gender (in-)equality; further socio-cultural references, e.g., lowest employment rates for women and low fertility rates in Italy compared to other European countries, Italian working and workplace culture (work-devotion schema, ideal worker model, Italy as conservative country with emphasis on presence at work by workplaces and employers).	Not reported;<br>Higher social class (educational attainment, employment status, socioeconomic condition) is considered as a "critical case" in that parents of higher social class have a) financial means to purchase social services on the labour market and b) more sociocultural capital / knowledge to challenge gender norms (critical case design at the intersection of parenthood, high education, and SES). However, social class is not analysed at the intersection to gender norms; if anything, working conditions or labour market opportunities (and working models and difference in expectations towards and by fathers and mothers) are being discussed at the intersection to gender norms.
Cheng et al., 2021	Part of objectives	Gender binary;<br>No further conceptualisation.	Not reported

Clemens et al., 2021	Neither	Gender binary;<br>No further conceptualisation.	Not reported
Czymara et al., 2021	Both	Gender as categorical variable (male, female, divers), but reported as binary variable due to small number of diverse persons in the sample;<br>Reference to "doing gender" theory, the constitution and accomplishment of gender; discussion of the persistence of 2000s "gender inequality", the slow pace of "gender convergence in paid and unpaid work", as well as gender and differential crises-impact on work; review of gender differences in unpaid work in Germany and other countries ("gender inequality at home", "gendered division of labour"); reference to mental load as "a theoretically relevant dimension of gender inequality that is largely overlooked in the literature" with focus on the "cognitive dimension of gender inequality".	Not reported
Giurge et al., 2020	Title	Gender binary;<br>No further conceptualisation.	Implicit;<br>Reference to low-income women (gender x SES) spending most of their time on unpaid care work and reproductive work leaving them "time-poor" and with little time or leisure activities. Mentioning of low-income women primarily tending to spend most of their time on necessities, leaving them "time poor" and with little time for leisure activities (literature review).
Hipp & Bünning, 2021	Both	Gender binary;<br>Reference to gender segregation within the German labour market and the gendered division of paid and unpaid work, the gender gap in working hours and time spent on unpaid care work; reference to "doing gender" theory.	Implicit;<br>Analyses of differences within gender groups regarding several working constellations (gender x work constellations) and parenthood (gender x parenthood).
Ohlbrecht & Jellen, 2021	Objectives	Gender as categorical variable (male, female, divers), but reported as binary variable due to small number of diverse persons in the sample;<br>Review of gender-specific differences in the impact of the pandemic (financial worries and burdens, income loss, increase in childcare duties, multiple burdens, adjustment of working hours for childcare); mentioning of the COVID-19 crisis as "amplifier of social and gender inequality".	Implicit;<br>Reference to the educational gradient in health being more pronounced for women than men (gender x education); Analysis of educational qualification in correlation with life

			satisfaction although disregarding gender differences in this point.
Xue & McMunn, 2021	Both	Gender binary;<br>Discussion of how the unequal division of unpaid care work may impact employment participation, pay and progression exacerbating the gender pay gap.	Not reported
Yerkes et al., 2020	Both	Gender binary (gender as covariate; female = reference);<br>Discussion of the impact of COVID-19 lockdown measures on gender (in-) equality (employment-related changes which are worse for women, reflecting a "complex gendered reality; COVID-19 lockdown measure may exacerbate existing gender inequalities e.g. by "reaffirming women's caregiving roles", at the same time they have the potential to reduce gender inequalities by forcing families to "(re)negotiate the division of childcare and/or household work"); Reference to the socio-cultural setting of the Netherlands: in European comparison, the Netherlands score high on gender equality indices in the domains of work, health, and knowledge, but there is gender inequality particularly in relation to paid work and (unpaid) care (work) with reference to Netherland's "one-and-a-half earner model".	Not reported
Zhou & Kan, 2021	Objectives	Gender binary;<br>Discussion of gender inequalities in terms of labour market participation (higher for men, more men in full-time in the UK) which are traced back to inflexible workplace expectations, gender norms (men as primary earners, women as primary caregivers) and labour market discrimination; discussion of gender inequalities in terms of COVID-19-related impacts on employment (men more likely to be furloughed, laid off, quit their jobs), and working mothers being more likely to reduce work hours or leave jobs; impacts on the division of unpaid care work with contradictory findings (improvement as increase in domestic work was larger for men vs. the contrary); impact on subjective wellbeing with differential decline for men vs. women.	Not reported
Grey literature			
Bolis et al., 2020	Title[2, 3]	Gender binary;<br>Reference to gender and economic inequalities caused by patriarchal and capitalist economic systems, and gender care gaps in the background section.	Not reported.

Bujard et al., 2020	Neither	Gender binary;<br>Discussion of the traditional concepts of motherhood (caring) vs. fatherhood in Germany: there is no re-traditionalisation with regards to unpaid care work division, since that would require a previous de-traditionalisation; as such, the COVID-19 pandemic has reinforced traditional division of unpaid care work within heterosexual parents (although not for all working arrangements) in Germany.	Implicit;<br>Analyses of differences in care and house work as well as family satisfaction within gender groups regarding working settings (working hours, setting, reduced working hours)
Chung et al., 2020	Objectives	Gender binary;<br>No further conceptualisation.	Not reported
Close the Gap & Engender, 2021	Both	Gender binary;<br>Review of gender inequality in terms of financial/ economic, labour-market related outcomes, caring responsibilities and mental health, especially at the intersection to ethnicity and (dis-)ability, both during and before the COVID-19 pandemic; mentioning of the principles of a "gender-sensitive economic recovery" as guide for post-pandemic gender-inclusive economic recovery strategy.	Explicit;<br>Mentioning of the need for not only integrating gendered perspectives but also an intersectional approach to policymaking with regards to mental health, labour market and economic recovery; discussion of a potential growing labour market inequality for BME and disabled women; results for anxiety levels are reported by gender (female/male) x ethnicity (BAME/white) as well as by gender (female/male) and disability (disabled/non-disabled); results for unpaid care work are reported by gender only.
Destatis, WZB & BiB, 2021	Neither	Gender binary;<br>No further conceptualisation.	Implicit;<br>Differences within gender groups for housework and care work regarding work settings (work settings x gender).
Etheridge & Spantig, 2020	Both	Gender binary;<br>Review of gender inequalities in mental health in the UK pre-pandemic.	Not reported
Eurocarers/ IRCCS-INRCA, 2021	Neither	Gender binary;<br>No further conceptualisation.	Not reported

Eurofound, 2020	Neither	Gender binary;<br>Mentioning of COVID-19 as being at risk to become a turning point for decade-long accomplishments of gender equality; review of pandemic-related gender equalities in terms of health risks, labour-market outcomes and caregiving responsibilities.	Not reported
Hübgen et al., 2021	Objectives	Gender binary;<br>Discussion of gender inequalities in terms of working conditions (e.g., statistics on short-term work, worktime reduction, unemployment/being furloughed, emergency aid) and family life (e.g., care work, work-life balance) during and before the COVID-19 pandemic. Survey on gender norms at two time points (June 2020, March 2021) on a scale from 1 = completely disagree to 7 = completely agree (question not available).	Implicit;<br>Intra-categorical differences within gender groups were referenced with regard to a more egalitarian care work division among academic parents compared to less educated parents (gender x education); explicit mentioning of intersectional approaches for further research.
Illing et al., 2022	Title	Gender binary;<br>Reference to gender inequalities in terms of working life and family life before and during the COVID-19 pandemic in the background section.	Not reported
Kalaylıoğlu et al., 2020	Both	Gender binary;<br>Reference to the persistence of the male breadwinner norm reinforcing women's traditional caregiver roles, notably reflected in the one of the highest unpaid care work burden for women in Türkiye among OECD countries.	Not reported
OECD, 2021	Title[2]	Gender binary;<br>Discussion of gender gaps in paid and unpaid work before and during the COVID-19 pandemic.	Not reported
Tani et al., 2021	Objectives	Gender binary;<br>No further conceptualization.	Implicit;<br>Differences within gender groups (mothers/fathers) regarding financial insecurity, income, and mental health were assessed (gender x income/ financial insecurity); people with a pre-pandemic low income, especially women, suffer from higher levels of mental and financial distress (discussion).

The Fawcett Society et al., 2020	Objectives	Gender binary;<br>Discussion of gender inequalities in terms of social, personal and economic outcomes at the intersection to ethnic minority backgrounds in the background section.	Explicit;<br>Intersectionally-conceptualised study mentioning the importance of intersections between gender and racialisation, socio-economic disadvantage, and disability.<br>Mentioning of intersections of gender and ethnic minority backgrounds (based on an understanding of racial differences being rooted in socio-economic inequalities linked to structural racism) as source for greater risks of physical, psychological, and financial impacts on BAME women;<br>Results for domestic and care work as well as for life satisfaction and happiness are reported by gender (female/male) x ethnicity (BAME/white).
Zoch, Bächmann & Vicari, 2020	Neither	Gender binary;<br>Discussion and survey of gender roles as driving force of systematic differences in the division of family work based on a) respondents' agreement with the statement "it's the man's job to earn money and woman's job to take care of the household and family" on a 4-point scale from 1 = completely disagree to 4 = completely agree; more traditional gender roles go hand in hand with lower paternal childcare involvement while increasing the likelihood of exclusive maternal care.	Implicit;<br>Differences in care-arrangements of gender groups regarding gender norms, working conditions and education (care-arrangements x education (college/university) x several working conditions (working hours, keyworker status, remote work) x gender norms (traditional roles)) were discussed.

Notes: BAME = Black, Asian and Minority Ethnicity. HE = Higher Education. HEI = Higher Education Institutions. SES = Socio-economic status.

[1]The degree to which intersectionality was mentioned in included articles and reports is divided into three: (1) explicit (= intersectionality was explicitly mentioned and interrelations/ intersections between gender and further social categories are analysed), (2) implicit (= intersectionality was not explicitly mentioned, however implied in that interrelations/ intersections between gender and further social categories were reported/ mentioned), and (3) not reported.

[2]In this report, no aims or objectives were mentioned.

[3]The title includes the word "feminist".
