# Supplementary material for: Caring is not always sharing: A scoping review exploring how COVID-19 containment measures have impacted unpaid care work and mental health among women and men in Europe
Source: PLoS One. 2024 Aug 30;19(8):e0308381. doi: 10.1371/journal.pone.0308381 (PMC11364293; doi:10.1371/journal.pone.0308381)
Supplement: S1 Appendix — (PDF) [file pone.0308381.s005.pdf]

Caring is not always sharing: A scoping review exploring how COVID-19 containment measures have impacted unpaid care work and mental health among women and men in Europe

S2 Appendix: Documentation of the grey literature search

Google Scholar

Search strings and search history

#	SEARCH STRINGS	RECORDS (13 May 2022)	EXTRACTED PAGES	SCREENED RECORDS
1	("unpaid care"|"informal care"|"family care"|caregiving|caregivers|carers|childcare|"child-care"|housework|"domestic work"|"unpaid work") (lockdown|shutdowns|quarantine|"containment measures"|"measures to contain"|COVID-19|coronavirus)	16,500	All 100	996
2	("work-life balance"|"work-family conflict"|"work-to-family conflict") (lockdown|shutdowns|quarantine|"containment measures"|"measures to contain"|COVID-19|coronavirus)	17,500	First 50 + 50 hand-searched	528
				**1,524**

Search strategy & a quick guide for systematic literature searches on Google Scholar

The search was performed separately, as only 256 characters (including space) were allowed per string. The references and citations of all 100 pages (10 records per page) were extracted, resulting in 996 records after excluding duplicates. The second search string was used as a supplement to string 1 to complete the main search (as these three key words did not fit into the first search string, but were found to be important after performing the sample search). Therefore, only the first 50 pages were extracted, and a further 50 pages were screened on Google Scholar to extract individual titles that HG considered interesting (n = 528 records). HG used Google Scholar's My library feature to first save the references and then perform a bulk export to Citavi Reference Manager (Swiss Academic Software, Switzerland). The records were screened for duplicates and then exported to Excel spreadsheets. The screening was performed using these Excel spreadsheets. As Google Scholar is very sensitive to potential malware, HG had to be careful not to make too many repeated requests and had to alternate between saving and extracting records (and deleting the saved records after extraction) to avoid a ban from Google Scholar. In a previous attempt, a bulk export tool (i.e., Zotero Reference Manager Connector for Windows Edge) was used, but the process was banned after Google Scholar reported potential malware. HG therefore recommends the above-mentioned approach.

Website search

Website searches were conducted by HG on 24 April 2022 (n = 137) and updated on 3 December 2022 (n = 158). HG clicked on the "Publications" section of each website and searched all relevant publication types for predefined keywords in titles and, where available, in abstracts. Additionally, HG searched for the predefined keywords in the search window of each website. Relevant documents were downloaded and their bibliography (i.e., authors, title, institution/ organisation, URL, publication type) was transferred into an Excel spreadsheet for screening.

**Lists of websites searched for grey literature and number of records identified**		
European Organisations	Coface EU https://coface-eu.org/	0
	Eurocarers https://eurocarers.org/	4
	European Commission https://ec.europa.eu/	2
	European Institute for Gender Equality (EIGE) https://eige.europa.eu/	4
	European Parliament https://www.europarl.europa.eu/	5
	European Union (EU) https://european-union.europa.eu/	0
	European Women's Lobby https://womenlobby.org/	0
International organizations	International Labour Organization (ILO) https://www.ilo.org/global/	3
	Organisation for Economic Co-operation and Development (OECD) https://www.oecd.org/	1
	United Nations (UN) https://www.un.org/en/	0
	UN Women https://www.unwomen.org/en	7
	World Health Organization (WHO) https://www.euro.who.int/en/home	0
Research institutes	Centre for Economic and Policy Research (CEPR) https://cepr.org/	7
	German Institute for Economic Research (DIW) https://www.diw.de/	14
	Institute for Employment Research (IAB) https://www.iab.de/	10
	Institute for Fiscal Studies (IFS) https://ifs.org.uk/	7
	Institute for Social and Economic Research (ISER) https://www.iser.essex.ac.uk/	7
	Institute of Labor Economics (IZA) https://www.iza.org/en	18
	Robert Koch Institute (RKI)	8

	https://www.rki.de/	
	Berlin Social Science Center (WZB) https://wzb.eu/de	3
	Institute of Economic and Social Research (WSI) https://www.wsi.de/	12
Think tanks/ NGOs	Bertelsmann Foundation https://www.bertelsmann-stiftung.de/	1
	Carers UK https://www.carersuk.org/	14
	Center for Intersectional Justice (CIJ) https://www.intersectionaljustice.org/	0
	Engender https://www.engender.org.uk/	3
	Equal Care Day https://equalcareday.de/	0
	Friedrich Ebert Foundation https://www.fes.de/	7
	Gunda Werner Institute https://www.gwi-boell.de/index.php/de	0
	Hans Böckler Foundation https://www.boeckler.de/	0
	Heinrich Böll Foundation https://www.boell.de/de	0
	Oxfam https://oxfamilibrary.openrepository.com/	4
Survey websites	Eurofound https://www.eurofound.europa.eu/	6
	Eurostat https://ec.europa.eu/eurostat/web/main/home	0
	Survey of Health, Ageing and Retirement in Europe (SHARE) http://www.share-project.org/home0.html	3
	University of Mannheim (Mannheim Corona Study) https://www.uni-mannheim.de/gip/corona-studie/	5
Corporate organizations	McKinsey Group https://www.mckinsey.com/	1
Government websites	Federal Ministry for Family Affair, Senior Citizens, Women and Youth (BMFSFJ) https://www.bmfsfj.de/bmfsfj	2
Total		158
